# A lightweight convolutional neural network architecture for violence detection in video sequences

**DOI:** 10.1038/s41598-026-37743-0

**Published:** 2026-02-06

**Authors:** Bhawana Tyagi, Richa Jain, Pankaj Jain, R. Naga Priyadarsini, Ashish Sharma

**Affiliations:** 1 School of Computer Science and Engineering, VIT University, Vellore, Tamil Nadu India; 2 Department of Computer Science, Banasthali Vidyapith, Niwai, Rajasthan India; 3 Department of Computer Science and Engineering, JECRC University, Jaipur, Rajasthan India; 4 Department of Electronics and Communication Engineering, Manipal Institute of Technology Bengaluru, Manipal Academy of Higher Education, Manipal, India

**Keywords:** Violence detection, Deep learning, Mobilenetv2, Convolutional neural network, Inceptionv3, VGG19, Engineering, Mathematics and computing

## Abstract

The escalation of violent incidents in high-density public environments such as political assemblies, concerts, and sports arenas necessitates the development of computationally efficient and accurate real-time violence detection frameworks. Prompt identification of aggressive events from continuous surveillance video streams is critical for initiating rapid countermeasures. However, the task is inherently complex due to spatiotemporal scene variations, illumination inconsistencies, and the intensive computational cost of processing high-dimensional video data. This study introduces a lightweight deep convolutional neural network (CNN) architecture derived from MobileNetV2, optimized through depthwise separable convolutions and inverted residual bottlenecks to achieve significant parameter reduction without compromising classification efficacy. The proposed framework processes video streams by extracting and preprocessing frames (224 × 224 resolution, normalization, augmentation) to enhance generalization and mitigate overfitting. The model was trained and evaluated on two benchmark datasets: the Real-Life Violence Situations Dataset (RLVSD) and the Hockey Fight Dataset (HFD), encompassing balanced classes of violent and non-violent sequences. Empirical evaluation indicates superior performance, attaining 97% accuracy on RLVSD and 94% on HFD, with corresponding gains in precision, recall, and F1-score compared to conventional CNN architectures. Computational profiling confirms substantial efficiency improvements, enabling inference at real-time frame rates on resource-constrained hardware. The proposed methodology demonstrates that optimized lightweight architectures can deliver high-accuracy violence detection while significantly reducing computational overhead. These characteristics make the approach highly deployable in real-world surveillance systems. Future research will focus on temporal feature integration via 3D CNNs or transformer-based models and cross-domain adaptability to heterogeneous video sources.

## Introduction

 In today’s scenario, terrorist attacks and crimes are arising day by day, which requires an intelligent surveillance system that can automatically identify these activities at the earliest by recognizing the behavior of the crowd. There are many places like railway stations, airports, malls, political rallies, colleges, etc. where surveillance is required for security purposes, as the chances of the occurrence of abnormal behavior increase with an increase in crowds. From past few years the installation of closed circuit television (CCTV) is increased enormously at various public and personal places. In the conventional surveillance methods, human operators are required to continuously monitor live videos, which is both tedious and error prone. Critical information is often overlooked due to fatigue or loss of concentration during prolonged monitoring. Consequently, there is a need for fully automated system capable of identifying abnormal crowd behavior and generating alerts when suspicious activities are detected. Hence, abnormal activity recognition has become a prevalent research area in recent years. Crowd behavior detection has numerous applications, including video surveillance systems, autonomous driving, and human-machine interaction^1–3^. Among these, violence detection is a major concern for many researchers nowadays. However, it persists as a major research problem due to aspects including varying illumination conditions, heterogeneous and noisy backgrounds, multiple camera orientations, heterogeneity in human form and posture, and occlusion, among others^4,5^.

Numerous studies^6–8^ have been conducted in this field in academia and industry worldwide. However, existing violence detection systems are computationally expensive and often limited in accuracy, with no method achieving both low cost and high accuracy. In real-time applications, there is a need for a system that are accurate, efficient, and capable of automatically detecting violent events in video streams. Prior to the advent of deep learning, many computer vision-based methods^9–12^ were proposed for detecting violent behaviors in videos, but the accuracy of these approaches was relatively limited. With the emergence of the deep learning era, numerous strategies have been proposed for real-time violent behavior detection. Nevertheless, labeling data as violent or non-violent remains challenging because certain behaviors are context-dependent. For example, aggressive actions such as boxing are considered normal in a sporting arena but would be classified as violent activity in a public setting.

The architecture proposed is inspired by MobileNetV2 but brings in several architectural and functional changes that are specific to video-based violence detection rather than merely indicating generic object classification. Its main distinguishing contributions are the following:

- Squeeze-and-Excitation (SE) attention integrated within each bottleneck block: This improvement permits the network to narrow down the attention to spatiotemporal motion patterns that are usually less pronounced during violent events and are perceptually standard in MobileNetV2.
- Layer-wise modification of expansion factors (t): The expansion ratios were reconfigured to strengthen high-level motion representation while reducing unnecessary channel inflation, resulting in better gradient stability on small-scale video datasets.
- A new lightweight convolution head: The 1280-channel head of MobileNetV2 is substituted with a 1440-unit head together with a dropout and a small classifier layer, thus the classification performance is bettered and the number of parameters is reduced.
- An activation and regularization strategy based on the task: SiLU activation and dropout (*p* = 0.25) have been implemented to make the system more tolerant to lighting variations, camera movements, and impairments which are likely to coexist in real-world violent scenes.
- The proposal for the computational efficiency improvement model is a 1.93 M parameter count which is less than both MobileNet (3.23 M) and MobileNetV2 (2.26 M), thus enabling the model to perform real-time inference on low-power devices.
- The architecture proposed is tested on the Hockey Fight Dataset (HFD)^13^ and Real-Life Violence Situations Dataset (RLVSD)^14^ to showcase its efficacy.

The remaining segments are ordered as follows: the literature review is covered in Sect. 2; Sect. 3 covers the background details along with description of dataset used and performance measures. The Sect. 4 comprises the description of proposed method. The suggested approach performance is discussed and compared to various available methodologies in Sect. 5. The Sect. 6 contains the conclusion and future work.

## Literature review

**Fig. 1:**
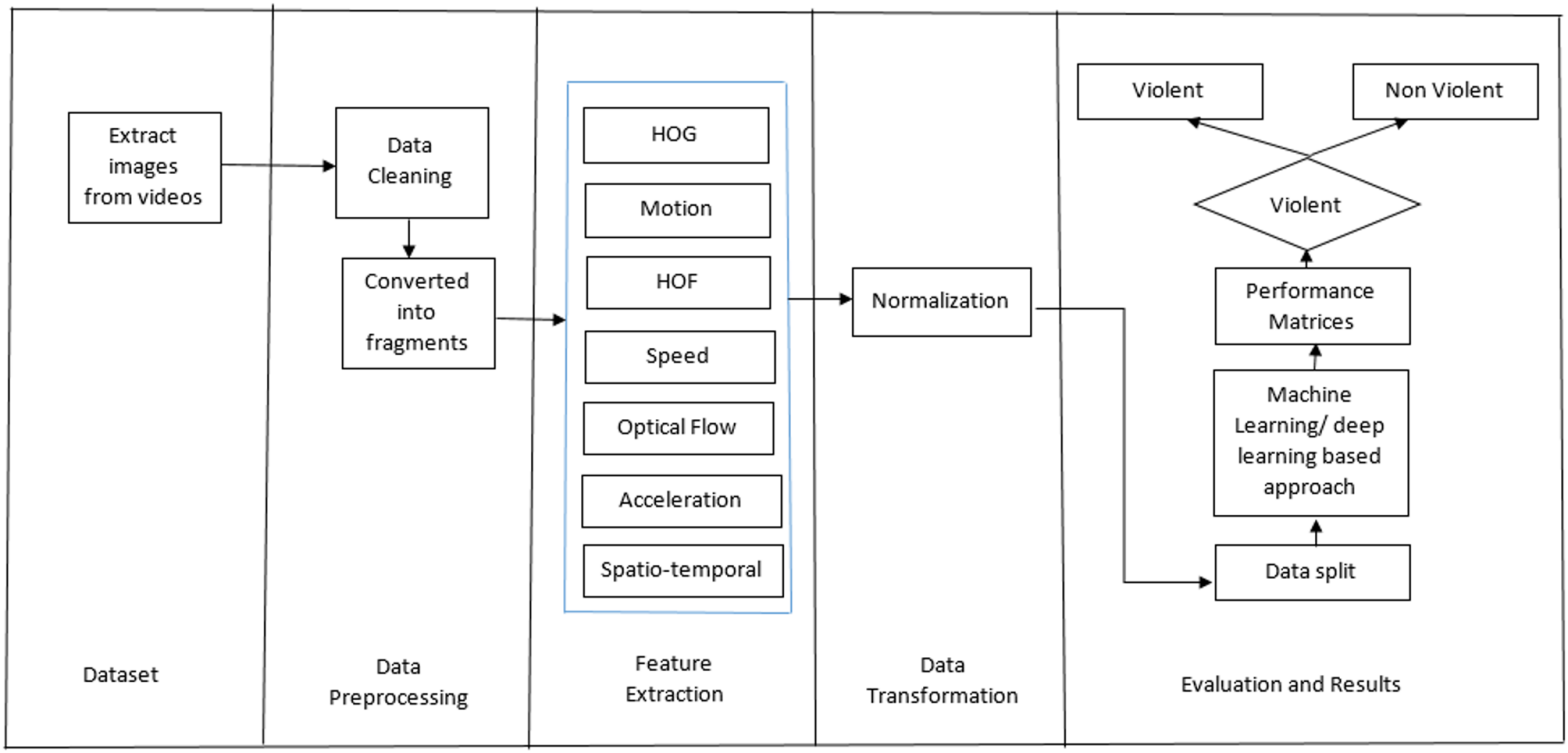
Basic steps to implement violence detection system^15^.

Numerous methods have been proposed in recent years and violence detection in the surveillance videos have been widely studied^15^. The basic workflow of a violence detection system is shown in Fig. 1. The generalized workflow begins with the frame extraction from the video sequence, followed by data pre-processing. In the next phase, features are extracted and converted into an appropriate feature space, which is then used for detecting anomalous or violent activities. These methods can be categorized in two parts: traditional techniques and deep learning techniques. The following subsections discuss these two categories in detail, outline the key research gaps, and finally present the research questions driving this work.

### Traditional methods

A machine learning based violence detection system is proposed by Penet et al.^16^ which utilized multimodal and temporal information. The MediaEval dataset is used for training and testing, and the system employed two-score learning algorithms. The system reports 3% missed detection and 50% false alarms. Another method is proposed by De Souza et al.^17^ that is proposed based on visual codebooks combined with a linear Support Vector Machine (SVM). Deniz et al.^18^ introduced another method in which exciting acceleration samples are used as a discriminative feature, achieving 12% higher accuracy in comparison with other available techniques.

Das et al.^19^ proposed a method in which low level features were extracted using Histogram of Oriented Gradient (HOG). Also, Several classifiers were evaluated, among which Random Forest achieved the higher accuracy of 86%. The improved violence detection framework was by Xu et al.^20^ presented improved framework which combined SVM and radial basis function (RBF) kernel. To extract low level features, they have used STIP and Kernel Density Estimation (KDE) was used to eliminate feature noise.

Violent flow variation was used by Arceda et al.^21^ to detect violence. It was based on the combination of Horn–Schunck method and SVM algorithm. The Horn–Schunck method performed better on Hockey fight dataset. Authors^22^ proposed cascaded method for violent behavior detection via movement filtering and motion boundary SIFT. The movement filtering was used to filter out all the samples that have nonviolent actions and further filtered frames were used for feature extraction. Another approach^23^ used Gaussian model and optical flow method to train SVM. A novel framework was proposed by Cai et al.^24^ that used complex trajectories with MPEG flow video descriptor and for feature encoding, fisher vector was used. A sparse representation was used by Cong et al.^25^ to find the abnormal behavior in crowds. Table 1 depicts the summary of the traditional methods used for violence detection.

**Table 1:** Summary of traditional methods for violence Detection.

Author(s) & Ref.	Approach / Method	Key Features / Techniques	Performance / Remarks
Penet et al.^16^	Bayesian network-based violent-shot detection using both audio and visual features integrated with temporal/contextual modeling.	Multimodal fusion; structure learning (Bayesian networks with K2 algorithm); audio/visual feature extraction; temporal context modeling.	Achieved ~ 50% false alarms and ~ 3% missed detections on the MediaEval 2011 dataset; multimodal and temporal integration shown to be effective; structural analysis yields interpretability.
De Souza et al.^17^	Shot-level violence detection using STIP (spatio-temporal) and SIFT features; BoVW representation; linear SVM classification.	STIP for motion detection; Bag of Visual Words; SVM classifier; comparison with spatial-only features (SIFT).	SIFT: ~80% accuracy on violent; STIP: ~99.5% accuracy on violent, 100% on non-violent. Highlights the critical importance of spatio-temporal cues.
Deniz et al.^18^	Kinematic cue–based violence detection via extreme accelerations, estimated through the Radon transform on frame-to-frame power spectra	Focus on extreme acceleration patterns; Radon transform on spectral motion representation; lightweight pipeline avoiding heavy feature extraction.	Up to 12% accuracy gain over generic action recognition; ≥15× faster runtime, enabling real-time viability.
Das et al.^19^	Frame selection (via subtraction and averaging), HOG feature extraction, followed by classification using various machine learning models (SVM, Random Forest, etc.).	HOG descriptors for capturing gradient and edge information; multi-algorithm comparison; lightweight and interpretable pipeline.	Best accuracy (~ 86%) achieved with Random Forest; demonstrates improvement vs. previous methods on benchmark dataset.
Xu et al.^20^	MoSIFT → KDE selection → Sparse coding → Max pooling → SVM	MoSIFT (appearance + motion), KDE-based feature selection, sparse coding, max pooling	≈ 93.6% accuracy—top-tier effectiveness for feature-based methods
Arceda et al.^21^	Real-time violence detection using the Violent Flow (ViF) descriptor—based on magnitude changes in optical flow vectors across time. Optical flow computed with Horn-Schunck, Lucas-Kanade, or IRLS; classification via SVM.	ViF captures abrupt motion changes; fast and lightweight; no preprocessing; evaluation of optical flow methods.	Outperforms LTP, HOG, HOF, HNF; competitive with STIP; accuracy ~ 90.9% (Hockey), ~ 89.5% (Movies); very fast, enabling real-time detection.
Febin et al.^22^	Movement filtering → MoBSIFT extraction → Classifier (RF, SVM, etc.)	Temporal derivative filtering, MoBSIFT (SIFT + optical flow + motion boundary), efficient cascade	Improved accuracy and computational efficiency; robust to camera movement
Zhang et al.^23^	GMOF for region localization → OHOF descriptors → linear SVM	Statistical modeling of optical flow deviations; OHOF captures motion orientation compactly; fast pipeline	Superior accuracy & speed vs. state-of-the-art; performs well in crowded scenes
Cai et al.^24^	DT / MF feature extraction → PCA → FV encoding → linear SVM	Rich spatio-temporal features; Fisher Vector preserves detailed statistics; optimized via PCA and K-means++	Outperforms other methods in accuracy and speed, effective in diverse scenarios
Cong et al.^25^	Sparse representation of local spatio-temporal cuboids; abnormality = high reconstruction error	Sparse representation of local spatio-temporal cuboids; abnormality = high reconstruction error	Sparse representation of local spatio-temporal cuboids; abnormality = high reconstruction error

### Deep learning based methods

To recognize different actions in the videos, Simonyan & Zisserman^26^ proposed CNN based model. They utilized spatial and temporal features by using separate networks for spatial and temporal. They have measured the performance on HMDB-51 and UCF-101 dataset. Bi-channel CNN and SVM based model^27^ selected the best features from different layers after comparison. To improve the accuracy, the motion and appearance information were combined. A triple staged deep learning-based method^28^ was based on spatiotemporal features. For automatic violence detection the 3D CNN trained model was optimized by using open visual inference. Three datasets were used, out of which the model outperforms on Hockey Fight dataset. Long short-term memory (LSTM) based method for violence detection is presented by Sudhakaran & Lanz^29^. The model received the subsequent frames difference as input to increase model accuracy.

A lightweight CNN based model was proposed by^30^, the model was optimized by using time domain filter to filter out the nonviolent scenes. The model reports 77.9% accuracy in case of violent class and reports 26.76% false positive rate on non-fight clips on ARENA and BEHAVE dataset, the model was not able to perform on the crowded video. The Visual Geometry Group (VGG)-19 CNN architecture was used by Butt et al.^31^ to extract features of objects. The major contribution was frame extraction from videos and object labelling. The Snatch 101 dataset was used for performance evaluation and model reports 81% accuracy. To detect violence in live video streams a 3D CNN was proposed by Accattoli et al.^32^. The new dataset consists of 350 videos proposed by Bianculli et al.^33^ to check the effectiveness of the violence detection techniques, the dataset includes 120 clips in nonviolent set and 230 videos in violent set. A multimodal information fusion based neural network was proposed by Pang et al.^34^, they have used bilinear pooling to fuse the audio and visual information. The method outperforms XD-violence dataset in comparison to other available methods.

In multi stream CNN^35^, temporal, spatio, and spatiotemporal features were fed to CNN. To learn the environmental patterns the spatial stream network was trained on every frame of the video, to study the motion pattern the temporal stream was used, and it considered 3 consecutive frames for that, and to represent violent action Spatio temporal stream was used. Hussain, et al.^36^ compared the effectiveness of various techniques on the HFD dataset. The authors compared AlexNet, VGG-16, GoogleNet and MobileNet architectures and reported that MobileNet outperformed in those architectures even with low computational cost. The robust deep neural network was proposed by Aarthy & Nithya^37^ by combining VGG 16 and LSTM. Initially the features were extracted using VGG 16 then extracted features fed to LSTM to detect violence in the video.

Researchers^38^ presents a three-stage deep learning framework for violence detection in surveillance videos. The model uses a lightweight convolutional neural network (CNN) to first identify individuals and filter out irrelevant frames. Next, a 3D-CNN extracts spatiotemporal features from a sequence of 50 frames, which are then classified as violent or non-violent using a SoftMax classifier. The study introduces a deep learning framework^39^ for anomaly detection in videos, addressing the challenge of generalizing models across diverse tasks without extensive retraining. The framework incorporates transfer learning, model fusion, and multitask classification, enabling it to generalize across multiple tasks with high accuracy. Empirical results show 97.99% accuracy on violence detection, 83.59% on shoplifting, and 88.37% across both. The framework also achieved strong performance on unseen datasets, offering a significant advancement in anomaly detection, ensuring fairness and robustness. Table 2 summarizes the deep learning based methods for violence detection.

**Table 2:** Summary of deep learning based methods for violence Detection.

Author(s) & Ref.	Approach / Method	Dataset Used	Key Features / Techniques	Performance / Remarks
Simonyan & Zisserman^26^	Two-Stream Convolutional Neural Networks (CNNs) for action recognition using separate spatial and temporal streams.	UCF101, HMDB51	- Spatial stream: RGB frames for appearance features. - Temporal stream: stacked optical flow for motion features. - Late fusion of softmax outputs. - Pre-training spatial stream on ImageNet. - Fine-tuning temporal stream on action datasets.	Achieved state-of-the-art accuracy at the time (UCF101: ~88.0%, HMDB51: ~59.4%). Influential architecture for later violence detection models using spatio-temporal deep features.
Xia et al.^27^	Real-time violence detection using deep spatio-temporal features extracted via a 3D CNN (C3D).	Hockey Fight Dataset, Movies Dataset	- C3D model captures spatial and temporal features jointly. - Input: sequences of consecutive frames. - End-to-end training for binary classification (violent / non-violent). - Optimized for fast inference.	Achieved high accuracy (~ 94–96%) with real-time performance (~ 25 fps). Robust against camera motion and background clutter
Ullah et al.^28^	3D Convolutional Neural Network (CNN) with spatiotemporal features	Benchmark violence detection video datasets (e.g., Hockey Fight, Movies dataset)	Extracts motion + appearance jointly using 3D CNN	Achieved high accuracy, robust against background noise and varying illumination
Sudhakaran & Lanz^29^	Convolutional LSTM (ConvLSTM) for sequence modeling	Violent Flows (ViF) dataset, Hockey Fight dataset	Temporal dynamics captured via ConvLSTM integrating CNN + LSTM	Outperformed hand-crafted features, strong temporal modeling
Baba et al.^30^	Sensor network + Deep Learning approach	Real-time smart city sensor data (video & IoT-based sensing)	Multimodal fusion from sensor networks with DL models	Suitable for smart cities, efficient real-time violence detection
Butt et al.^31^	VGG19 CNN for video frame classification	Video surveillance datasets (e.g., UCF-Crime, self-collected CCTV)	Transfer learning with deep pretrained CNN (VGG19)	High accuracy, computationally expensive but effective for surveillance
Accattoli et al.^32^	3D CNN features + Support Vector Machine (SVM) classifier	Public datasets (Hockey Fight, Movies, etc.)	Hybrid approach: feature extraction (3D CNN) + classification (SVM)	Improved classification accuracy vs. CNN alone
Bianculli et al.^33^	Dataset creation for automatic violence detection	Newly introduced dataset for violence detection in videos	Annotated violence vs. non-violence samples	Publicly available benchmark dataset, aids reproducibility and fair comparison
Pang et al.^34^	Fusion of visual + audio streams for violence detection	Public benchmark datasets with both video & audio (e.g., Hockey Fight, Movies)	Multimodal fusion (CNN for vision + spectrogram-based features for audio)	Outperformed visual-only models, highlighting audio’s role in violent scene recognition
Mohtavipour et al.^35^	Multi-stream CNN combining deep features + handcrafted features	Standard violence datasets (e.g., Hockey Fight, Violent-Flows)	Hybrid learning (CNN + handcrafted motion/texture descriptors)	Improved accuracy and robustness, especially on small datasets
Hussain et al.^36^	Real-time CNN-based violence detection framework	Surveillance video datasets (self-collected + public CCTV)	Lightweight CNN architecture optimized for speed	Achieved real-time performance with good accuracy, suitable for deployment
Aarthy & Nithya^37^	Deep learning architecture for crowd violence detection	Crowd violence video datasets (e.g., UCF-Crime, UMN)	CNN + sequence modeling for crowd behavior recognition	Effective in dense crowd scenarios; high accuracy but computationally intensive
Khan et al.^38^	Deep learning model for industrial surveillance	Industrial surveillance datasets (factory/warehouse environments)	Customized CNN for domain-specific violence patterns	High accuracy in industrial context; shows domain adaptation capability
Jebur et al.^39^	Scalable generalized deep learning framework for anomaly detection (violence included)	Large-scale surveillance datasets (multi-domain)	General anomaly detection via deep CNN + scalability mechanisms	Strong generalization; framework can detect violence + other anomalies across diverse settings

### Research gaps

- Need for automated feature extraction that captures both spatial and temporal patterns without manual engineering.
- Need for lightweight yet accurate architectures suitable for real-time surveillance systems.
- Need for models trained and evaluated on multiple heterogeneous datasets to improve robustness.
- Need for models incorporating scene context and motion filtering to improve precision.
- Need to design systems that can integrate with practical applications and support decision-making in real time.

### Research questions

RQ1: How can deep learning architectures be constructed to effectively extract spatial and temporal features from videos for precise violence detection?

RQ2: Is it possible to design a lightweight but resilient architecture that is both highly accurate in detection and computationally light enough for real-time deployment?

RQ3: How does the proposed model generalize when tested on multiple real-world violence datasets (e.g., RLVSD, HFD) with different environments and conditions?

RQ4: What techniques can be used to minimize false positives in crowded, complex, or noisy video scenes?

RQ5: How can the results be used in real-world surveillance systems to improve public safety and early threat detection?

## Background details

### Overview of Mobilenetv2

**Fig. 2:**
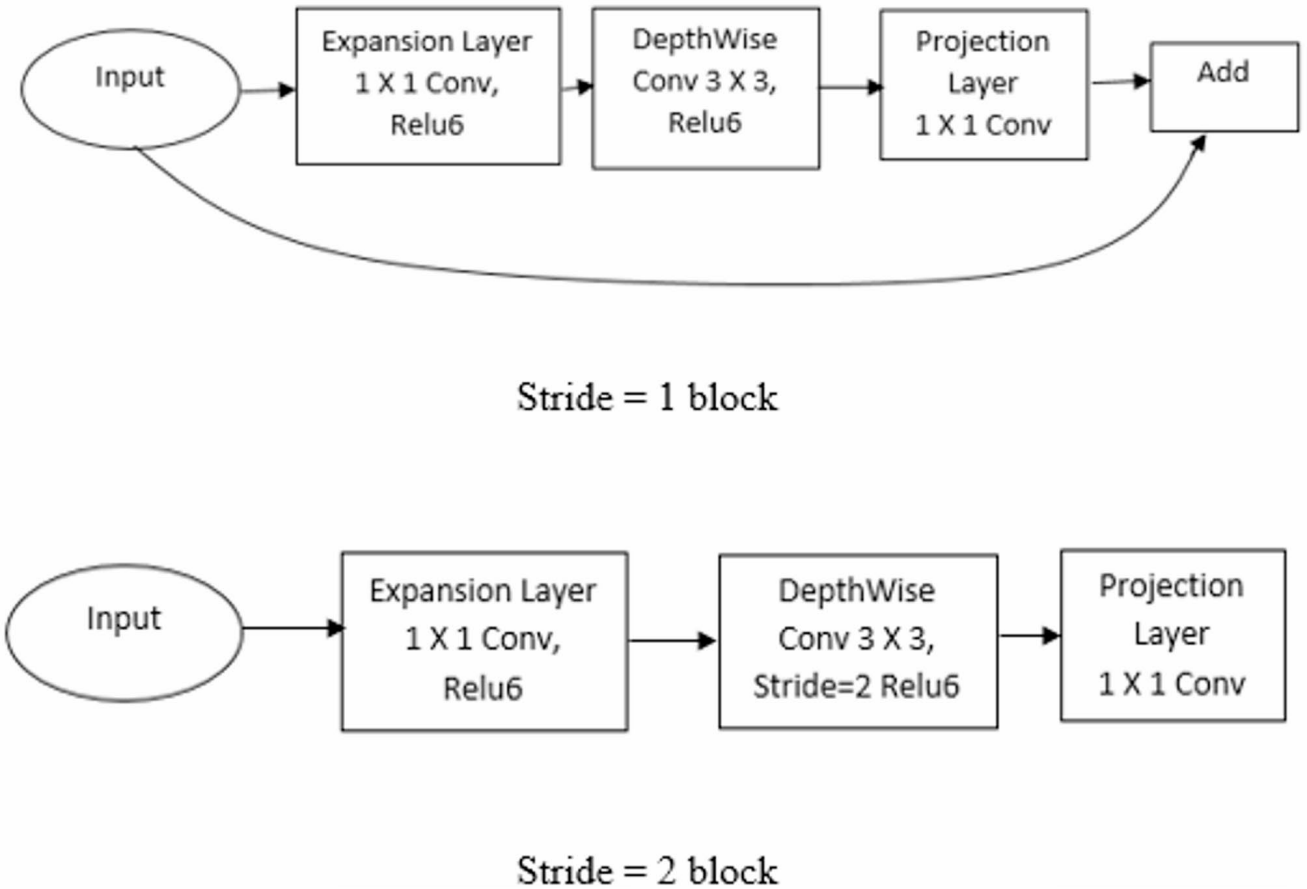
Mobilenetv2 Architecture.

Sandler et al.^40^ proposed Mobilenetv2, a CNN model with 53 layers. It is made up of two parts: an Inverted Residual Block (IRB) and a Bottleneck Residual Block (BRB). There are two kinds of convolution layers in it: $\:1\times\:1\:$convolution and $\:3\times\:3\:$depth wise convolution. One block is with stride 1, and the other is with stride 2 for down sampling. Both blocks have 3 convolution layers: first is expansion layer, second is depth-wise convolution layer and third is projection layer. The architecture of Mobilenetv2 is shown in Fig. 2. When stride 1 is used then the bottlenecks have the residual connection whereas when the stride is 2, the bottleneck does not have the residual connection. The expansion layer enlarges the data (total of channels increases) which passes through it. It is working just contrary to the third layer as the data gets expanded depending on the Expansion Factor (EF) used. By default, EF is 6. In second layer, for each input channel, a single convolution filter has been applied. The third layer decreases the volume of data which passes through it.

### Datasets

#### Real life violence situations dataset (RLVSD)

There are 2000 videos in this dataset. This dataset is divided into violent and non-violence video sets. There are 1000 videos in each set. These videos were collected from YouTube. Violence video contains fight scenes in different conditions and environment whereas non-violence videos contain simple actions like walking, playing, singing, eating, etc.

#### Hockey fight dataset (HFD)

Hockey fight dataset was proposed to provide more data that can be used for the fight detection system. There are 1000 video clips, divided into two sets. The first set contains 500 videos that are fight based whereas the other set contains 500 non-violent videos. Fight based sample images are shown in first row and the non-fight based images are shown in second row. In the dataset, there are 50 frames in each clip. The resolutions of the frames are $\:360\times\:288\:$pixels. Table 3 depicts the description of both the dataset.

**Table 3:** Dataset Description.

Dataset	Total Videos/Clips	Violent Videos	Non-Violent Videos	Source	Characteristics
Real Life Violence Situations Dataset (RLVSD)	2000 videos	1000	1000	YouTube	Violent set contains fight scenes in varied environments; non-violent set includes normal activities (walking, playing, singing, eating, etc.)
Hockey Fight Dataset (HFD)	1000 clips	500	500	Hockey game footage	Each clip has 50 frames (360 × 288 px); violent set contains fight scenes, non-violent set contains regular gameplay

### Performance measures

We have used the True Positive ($\:\mathcal{T}$P) rate, False Positive (FP) rate, True Negative ($\:\mathcal{T}$N) rate, False Negative (FN) rate, precision, recall, and accuracy to evaluate the performance of suggested model. The formula of precision shown in Eq. 1, recall is presented in Eq. 2, and Eq. 3 represents the formula of accuracy.

1 $$\:Precision=\:\frac{\mathcal{T}P}{\mathcal{T}P+FP}$$

2 $$\:Recall=\:\frac{\mathcal{T}P}{\mathcal{T}P+FN}$$

3 $$\:Accuracy=\:\frac{\mathcal{T}P+\mathcal{T}N}{\mathcal{T}P+FN+FP+\mathcal{T}N}$$

The $\:\mathcal{T}$P refers to those cases where both the video’s label and model indicate the presence of violent content. In instances where the model predicts a video to be non-violent and there is no evidence of violence depicted in the video, it is labeled as $\:\mathcal{T}$N. FP stands for the scenarios in which the algorithm anticipates that a video is violent even if it does not depict any instance of violence. FN refers to situations where the model interprets the video as free of violence when, in fact, it contains instances of violent content. The accuracy is the ratio between total numbers of correctly predicted values to the total number of predictions made.

## Proposed methodology

### Proposed light weight CNN model based on Mobilenetv2

**Fig. 3:**
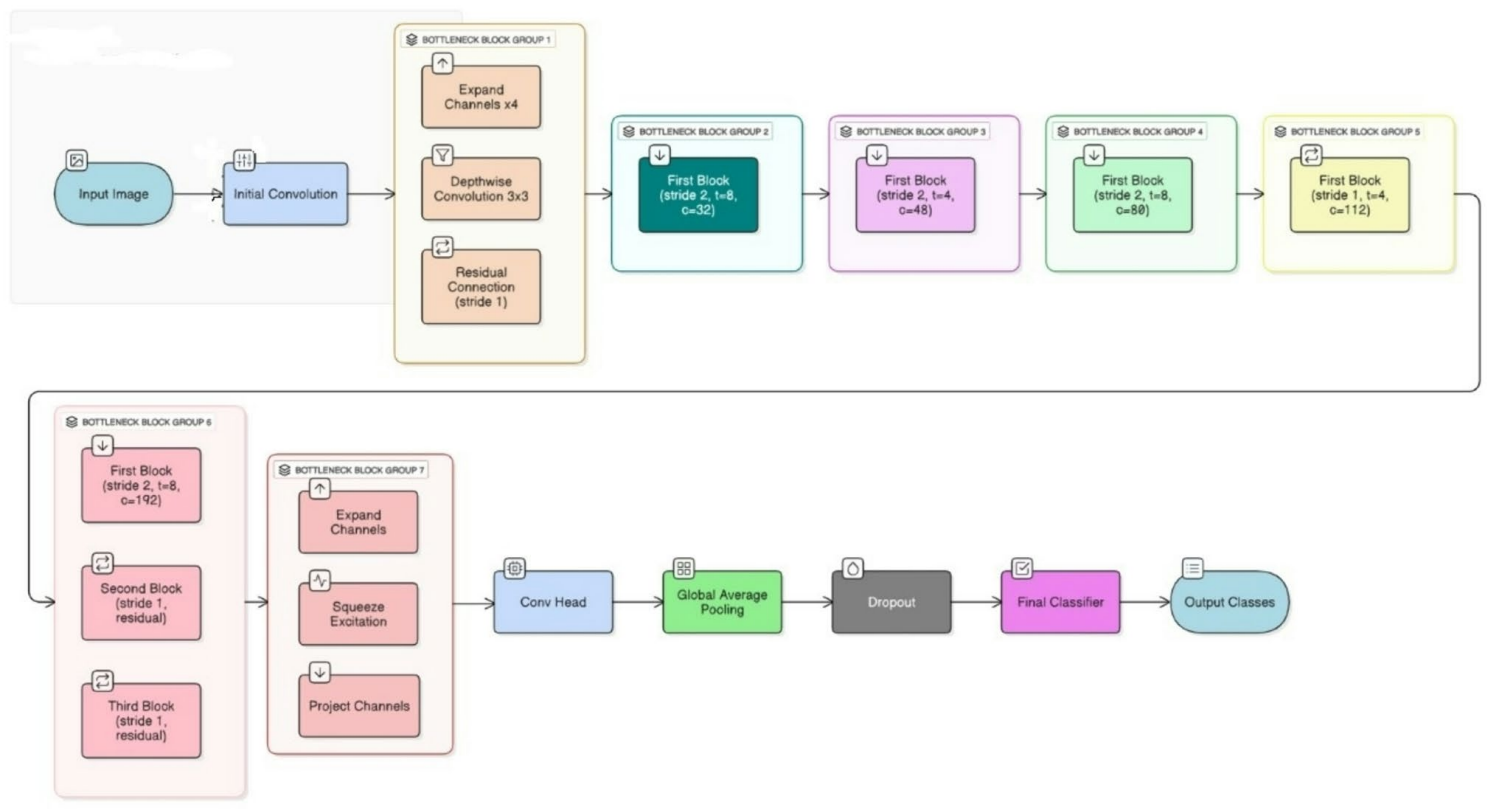
Architecture of proposed light weighted CNN model based on Mobilenetv2.

The basic architecture of the proposed method is the same as Mobilenetv2. There are two key components of this model: IRB and BRB. There are three layers in each block and two types of convolution layers used i.e., $\:1\times\:1$ and$\:\:3\times\:3$. One block has stride 1 and the other block has stride 2. The bottleneck has a residual connection when there is a stride is 1 and when the stride is 2 then there is no residual connection in the bottleneck. A complete detail of the network parameters is depicted in algorithm 1 and Fig. 3, t is the expansion factor, *c* denotes total of output channels, *n* denotes total number of repetitions, and *s* denotes strides. For spatial convolution, we have used $\:3\times\:3$ kernels. The Input tensor is X∈R^H×W× C^ ; and output Y will be Y∈R^H′×W′×C′^.

For a convolutional layer: kernel size k, stride s, padding p. Output spatial size H’ is given is Eq. 4 and W’ is given in Eq. 5.

4 $$\:{H}^{{\prime\:}}=\left\lfloor\left(\frac{H+2p-k}{s}\right)\right\rfloor+1$$

5 $$\:{W}^{{\prime\:}}=\left\lfloor\left(\frac{W+2p-k}{s}\right)\right\rfloor+1$$

The architecture starts with a starting convolution layer with a 3 × 3 kernel, stride 2, and 32 output channels followed by batch normalization (BN) and SiLU activation function and gives an output of 112 × 112 × 32. The input tensor: Y$\epsilon$R^224 × 224 × 3^ given to the starting layer which produces the output Y$\epsilon$R^112 × 112 × 32^. The detail expression Y is given in Eq. 6, which is followed by batch normalization BN(a_c_) given in Eq. 7 where µ _c_ is mean and σ_c_ is variance (per channel) used in Batch Normalization and SiLU activation given in Eq. 8.

6 $$\:{Y}_{i}{,}_{j}{,}_{c}=\sum\:_{u,v}\sum\:_{d=1}^{3}{W}_{u,v,d,c}^{\left(0\right)}{X}_{i+u,j+v,d}+{b}_{c}^{\left(0\right)}$$

7 $$\:BN\left({a}_{c}\right)={{\upgamma\:}}_{\mathrm{c}}\frac{{a}_{c}-{{\upmu\:}}_{c}}{\sqrt{{{\upsigma\:}}_{c}^{2}+{\upepsilon\:}}}+{{\upbeta\:}}_{c}$$

8 $$\:SiLU\left(x\right)=\frac{x}{1+{e}^{-x}}$$

Then, a bottleneck block with squeeze-and-excitation (SE) of expansion factor 4, output channels 24, one repetition, and a stride of 1 is used, which expands the input, does depthwise convolution, uses SE attention, projects back, and adds a residual connection. This results in an output of 112 × 112 × 24. The next block is another bottleneck block with SE, expansion factor 8, output channels 32, twice repeated, where the first one has stride 2 to decrease the size to 56 × 56 × 32 and the second one has stride 1 along with residual connection. The subsequent bottleneck block contains SE, expansion factor 4, output channels 48, three times repeated, with the first reducing size to 28 × 28 × 48 and the rest maintaining it. There is another bottleneck block with SE, expansion factor 8, output channels 80, repeated three times, reducing to 14 × 14 × 80 in the first block and then keeping size the same. There is then a bottleneck block with SE, expansion factor 4, output channels 112, repeated twice at 14 × 14 resolution. Next, a bottleneck block with SE, expansion factor 8, output channels 192, repeated three times, is used, where the first one downsizes to 7 × 7 × 192. One bottleneck block with SE, expansion factor 4, and output channels 256 follows, expanding, applying SE, and projecting but without downsizing. The network proceeds to the convolution head, where it employs a 1 × 1 convolution with 1440 output channels, BN, and SiLU to produce 7 × 7 × 1440. Global average pooling lowers this to 1 × 1 × 1440, to which is added dropout of 0.25 probability. Lastly, the classifier applies a 1 × 1 convolution with k output features, flattens this output, and places a softmax to deliver the final output.

**Algorithm 1:**
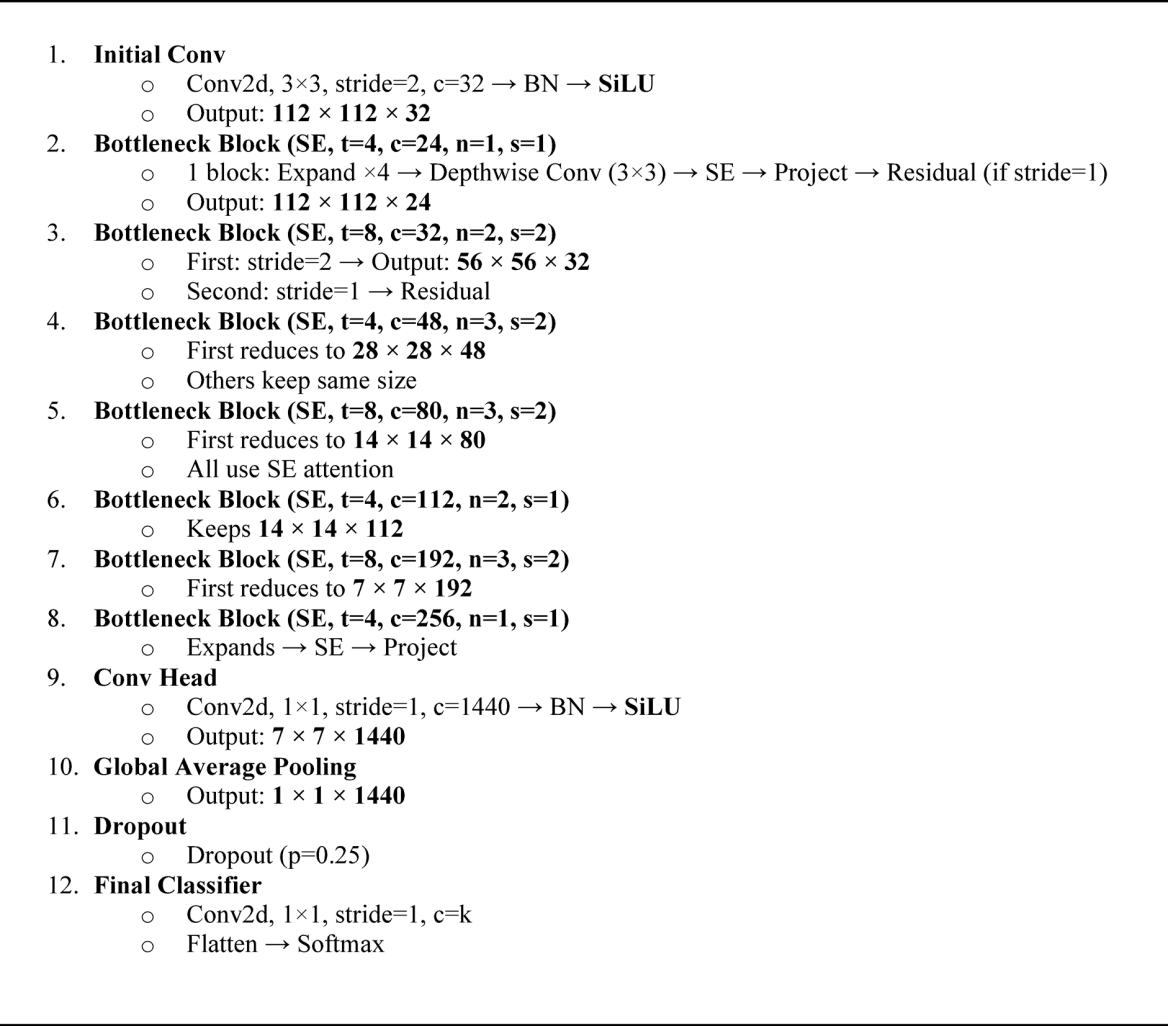
Proposed Violence Detection Algorithm.

### Proposed framework for violence detection

**Fig. 4:**
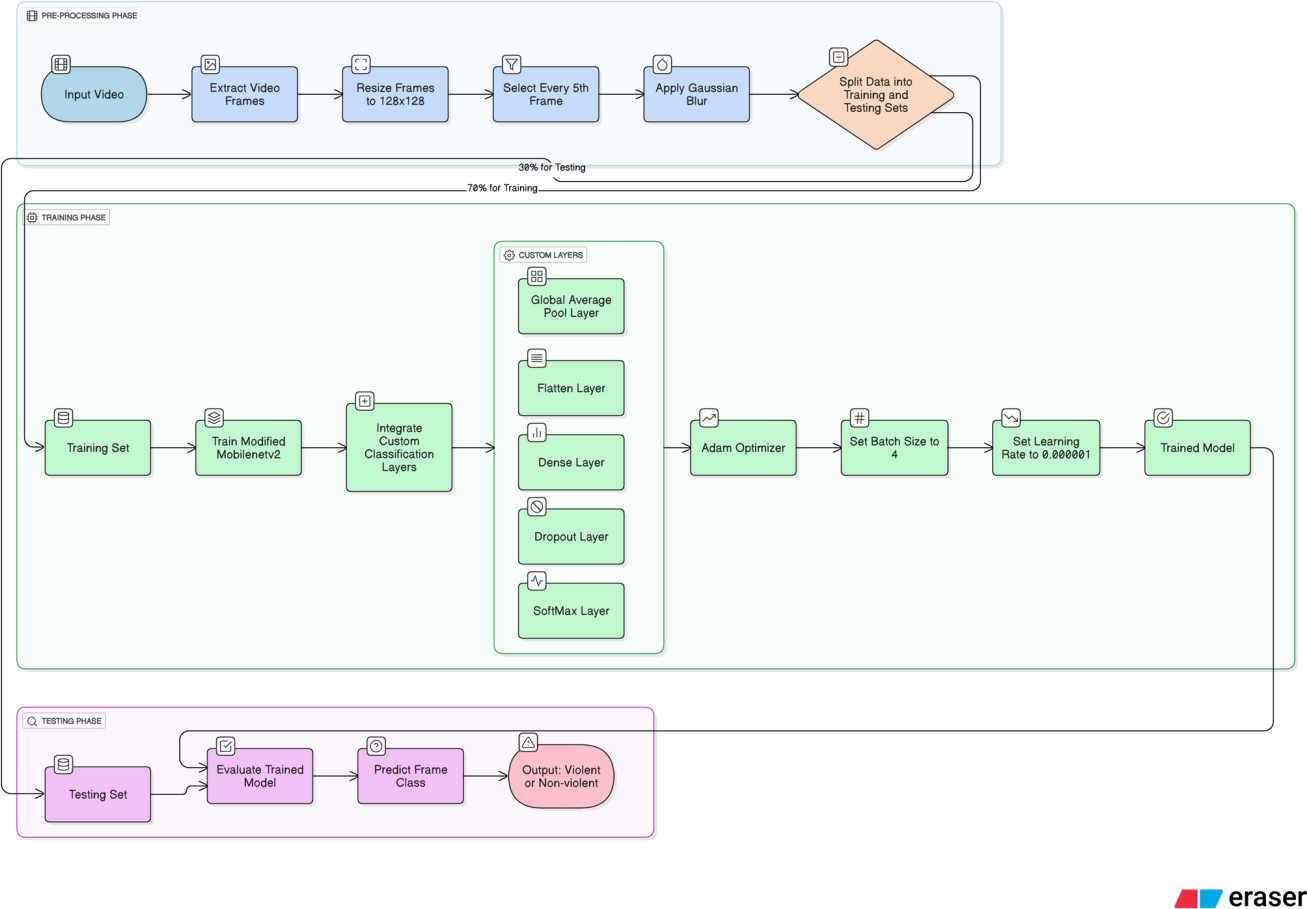
Proposed framework to detect violence.

The suggested framework has three main phases- preprocessing, training, and testing. Initially the frames are extracted from the videos and undergoes to preprocessing. After preprocessing, the proposed light weight CNN model is trained subsequently followed by testing. The suggested framework is presented in Fig. 4.

i. Pre-processing Phase.

This phase plays an essential job in utilizing the resource efficiently. It is applied to the video just before passing to the proposed model. As it is very complicated to process a massive amount of data. So, by using this step, the input video is processed. The video frames are taken out and reduced in size to$\:\:128\:X\:128$. Every 5th frame was taken to process just to eliminate the repetitions in the consecutive frames. To eliminate noise from the frames, we have used Gaussian blur. We have split the data into 70:30 ratio, where for training 70% is utilized.

From the video, every 5th frame was picked as the consecutive frames in the surveillance video were deeply correlated and thus, did not bring in any useful temporal variation. The sampling technique used here is a very efficient one; it cuts down on the number of frames but still retains the sudden motion patterns that are typical of violent events. The frames were made smaller to 224 × 224 pixels in order to conform to the spatial dimensions of the ImageNet pretraining, thus making it possible to do transfer learning effectively while keeping a small memory footprint. The Gaussian blur was applied to the images in order to get rid of the background noise and the undesired edges so that the depthwise convolution layers would extract the movement patterns instead of the texture artifacts. The final steps included normalization as well as applying data augmentation techniques, e.g. horizontal flip, rotation, and random shift, in order to increase generalization to various environments and camera viewpoints.

ii. Training Phase.

The pre-processed frames are given to train the modified Mobilenetv2. To train the models 70% dataset is used and to test and validate 30% dataset is used. We have used python as a simulation tool. The Keras and TensorFlow are used to set the implementation environment. We have used^13,14^ datasets. The batch size is 4. Adam optimizer^41^ is used to optimize the network parameters with 0.000001 learning rate. The hyperparameters as given in Table 4, were set based on preliminary experiments and literature-supported heuristics. To avoid divergence, a learning rate of 1 × 10^-6^ was employed during fine-tuning on small video datasets. The Adam optimizer was preferred in this case due to its stable convergence in input domains that are noisy and characterized by abrupt object motion. The batch size of 4 indicates a memory-efficient setting for high-resolution video tensors while still avoiding unstable gradients associated with smaller batch sizes. The dropout rate of 0.25 allows regularization without the loss of discriminative features, which is essential in violent event recognition where motion patterns might be short-lived. Early stopping was one of the measures taken to avoid overfitting during a long period of training.

iii. Testing Phase.

The model is prepared for testing once it has been trained. To evaluate the trained model, a test dataset will be used. The allocation of the test and train datasets is random, i.e., random images have been taken from the dataset for testing the model. The model will predict whether the frame is violent or nonviolent.

**Table 4:** Hyper parameters details.

S.No.	Hyper parameters	Values
1	Epochs	150 (with early stop)
2	Batch Size	4
3	Learning rate	0.000001
4	Loss	Categorical Cross entropy
5	Dropout	0.25
6	Optimizer	Adam

## Results and discussions

Here, we have provided a thorough comparison analysis of the proposed model and the existing models which are Inceptionv3, VGG-19, MobileNet, and Mobilenetv2. The proposed model results have been assessed with these models using quantitative metrics.

### Performance of the proposed model

**Fig. 5:**
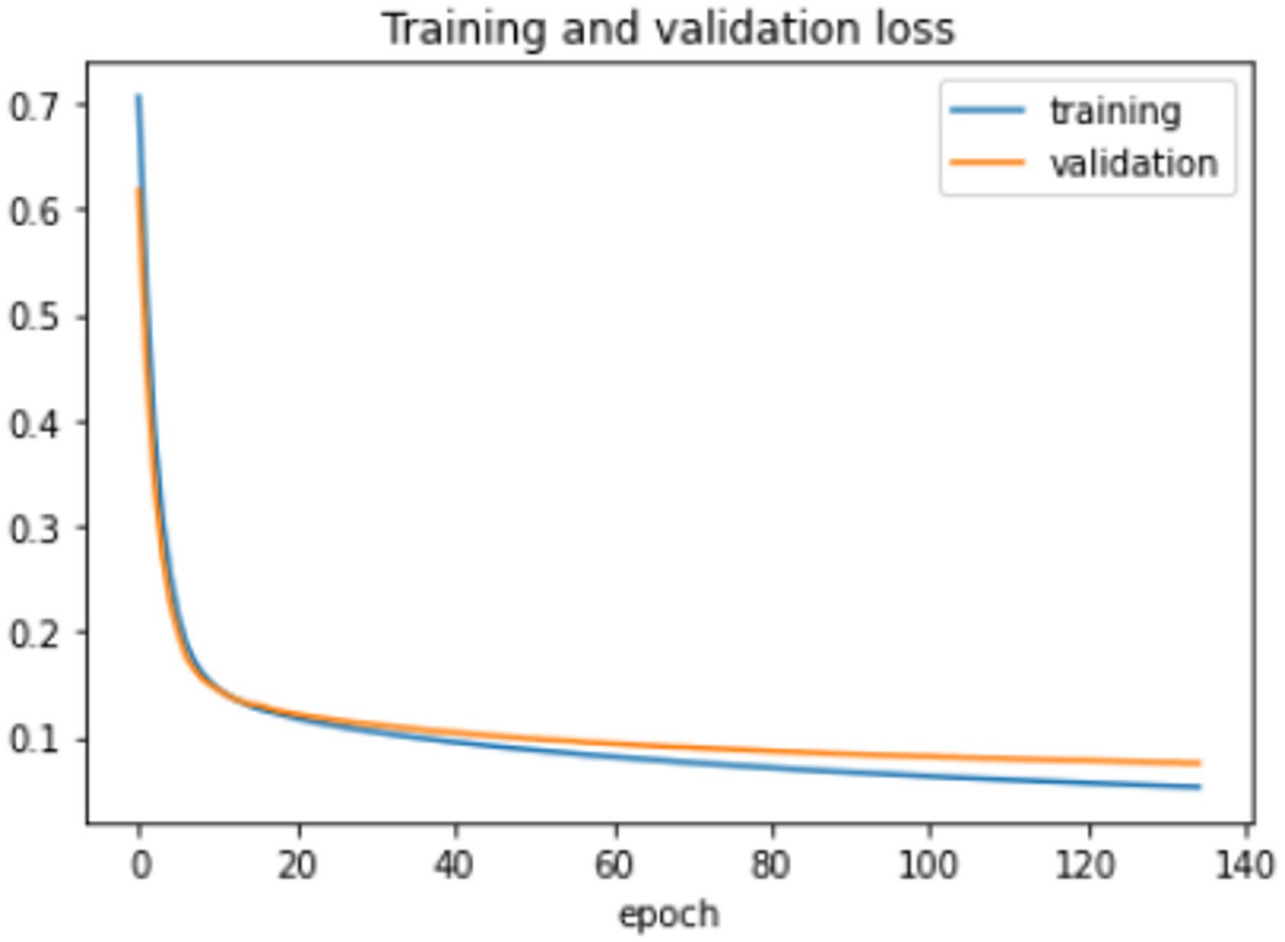
Training loss progress of proposed model on RLVSD.

**Fig. 6:**
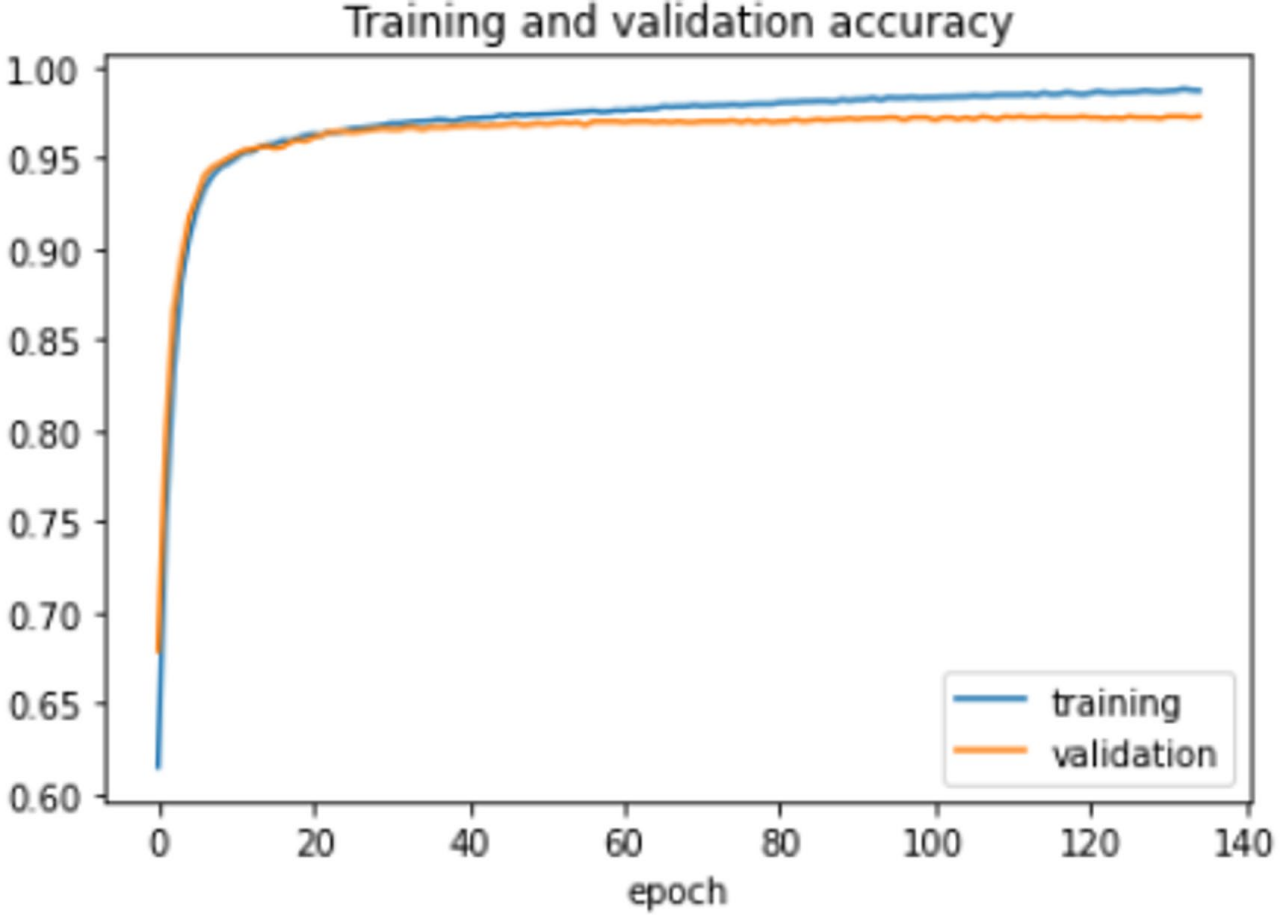
Accuracy progress of the proposed model on RLVSD.

The proposed model was trained for 150 epochs using the RLVSD with a learning rate of 0.00001. The proposed model performs well with a lesser computation parameter. The proposed light weight CNN model records fair accuracy on this dataset i.e., 97% accuracy for violence as well as non-violence class. Table 5 depicts the other performance metrics on both classes of dataset. Figure 5 shows the training loss progress and Fig. 6 illustrates the accuracy progress of the proposed model on this dataset.

**Table 5:** Performance metrics of proposed model on RLVSD.

	Precision(*P*)	Recall(*R*)	F1-Score(FS)
Nonviolence	0.98	0.97	0.97
Violence	0.97	0.98	0.98

**Fig. 7:**
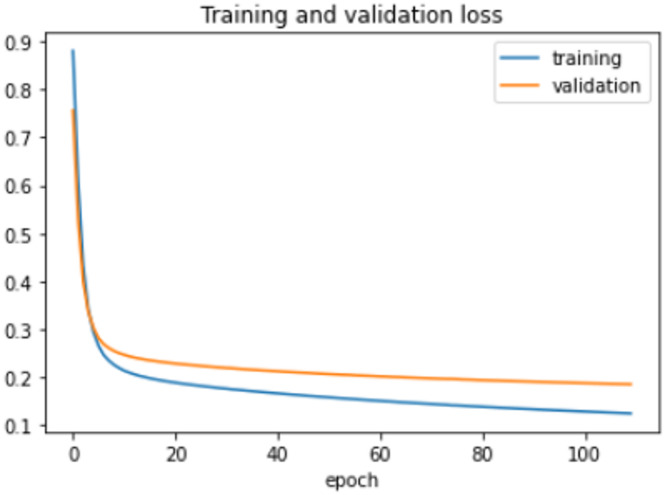
Training loss progress of proposed model on HFD.

**Fig. 8:**
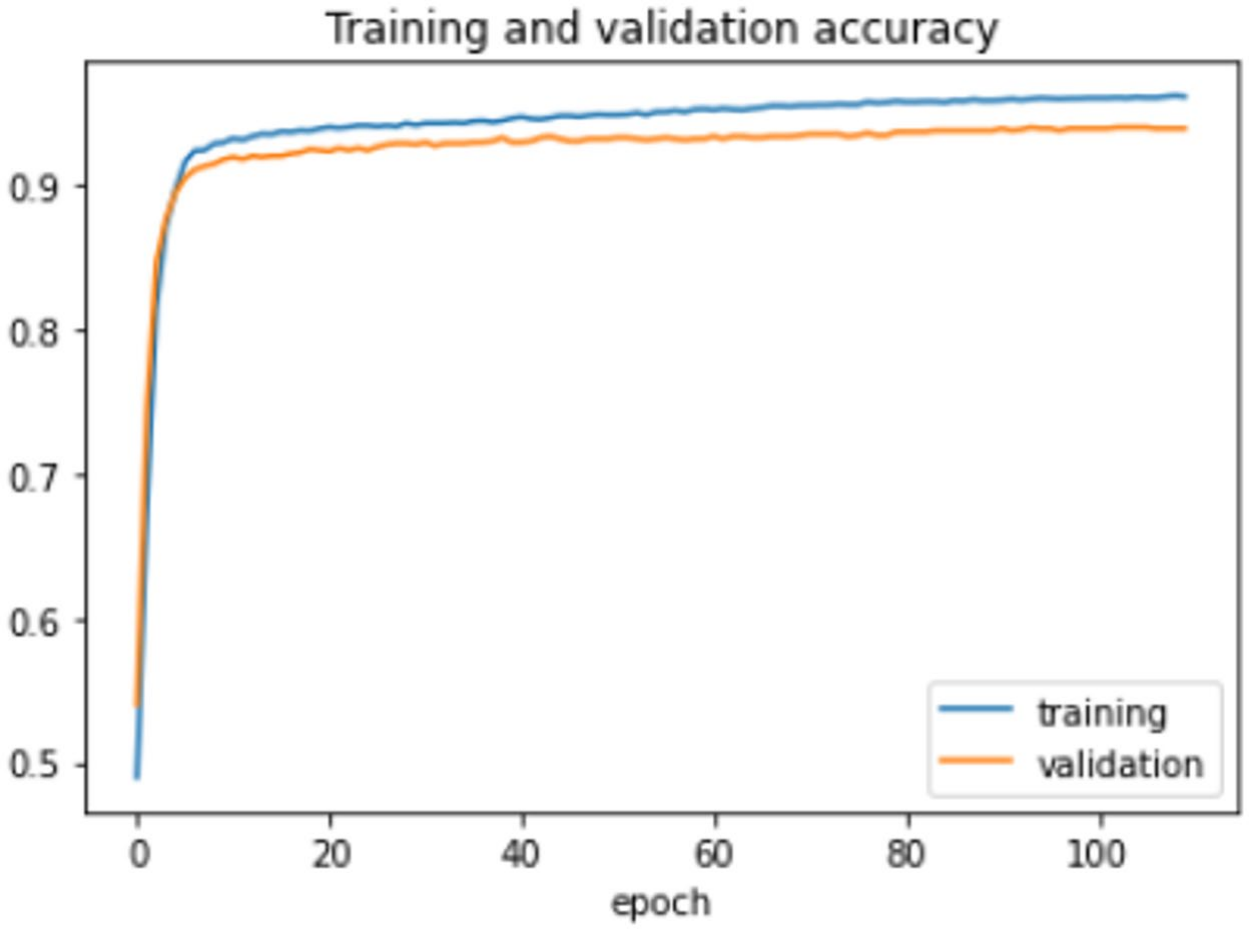
Accuracy progress of the proposed model on HFD.

On the HFD, the proposed model is trained for 150 epochs with the learning rate 0.000001. It reports 94% accuracy for non-violence class as well as violence class. For both the non-violence and violence class, the other performance metrics are recorded in Table 6. The training loss progress is depicted in Fig. 7 and accuracy progress of the proposed model on this dataset is shown in Fig. 8.

**Table 6:** Performance metrics of proposed model on HFD.

	*P*	*R*	FS
Nonviolence	0.94	0.94	0.94
Violence	0.94	0.94	0.94

### Performance of the Inceptionv3 model

**Fig. 9:**
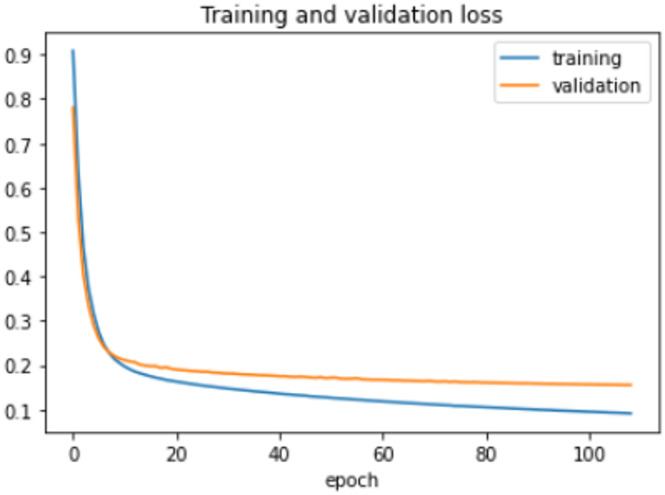
Inceptionv3 model’s training loss progress on RLVSD.

**Fig. 10:**
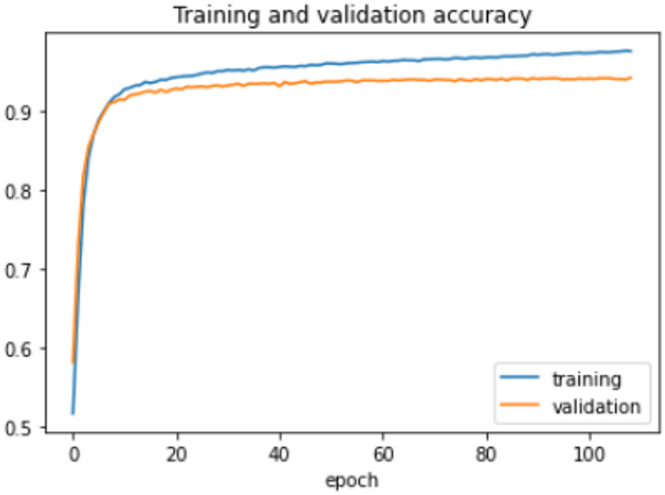
Accuracy progress of the Inceptionv3 model on RLVSD.

The Inceptionv3 Model^42^ is deep learning-based CNN model. It is used for classification purposes. It was proposed by Google. It has 42 layers. They have used factorization of large convolution into small to reduce the number of computational parameters. It has high efficiency in comparison to its previous version. The Inceptionv3 model was trained using 150 epochs and a learning rate of 0.000001 on the RLVSD. The best epoch was found at 109. The accuracy on the train is 97.58% whereas the accuracy on the test is 94.08%. The loss on the train is 0.09067 and on the test is 0.15471. The other performance metrics for violence and non-violence class are shown in Table 7. Figure 9 depicts the training loss progress and Fig. 10 depicts the accuracy progress of Inceptionv3 model’s on the RLVSD.

**Table 7:** Performance metrics of InceptionV3 model on RLVSD.

	*P*	*R*	FS
Nonviolence	0.95	0.92	0.94
Violence	0.93	0.96	0.94

**Fig. 11:**
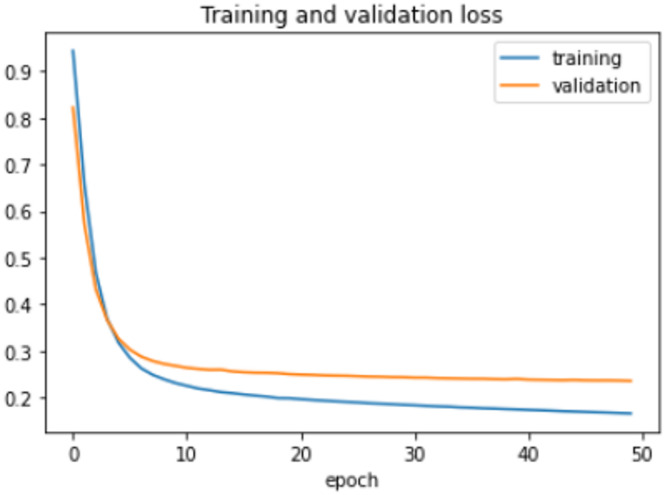
Inceptionv3 model’s training loss progress on HFD.

**Fig. 12:**
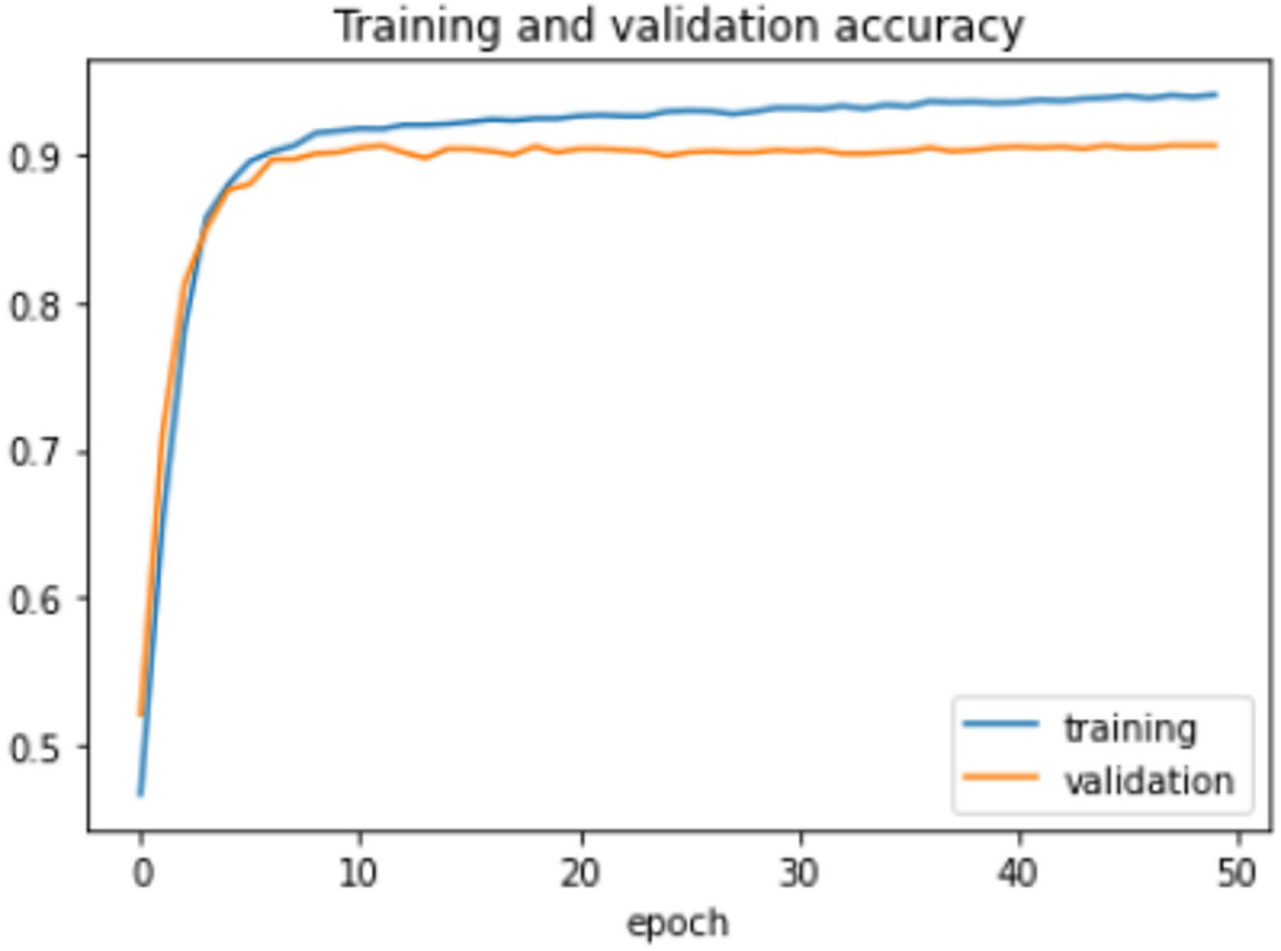
Accuracy progress of the InceptionV3 model on HFD.

The InceptionV3 model is trained for 150 epochs on the HFD with a learning rate of 0.000001. The best epoch is 50. The accuracy on the train is 94.05% whereas accuracy on test is 91.05%. The loss on the train is 0.165100 and on test is 0.22574. The other performance metrics are shown in Table 8, for nonviolence class the precision and F1-score are 0.91, and recall is 0.90. For the violence class the precision and F1-score is 0.91, and recall is 0.92. The Fig. 11 represents training loss progress and Fig. 12 represents accuracy progress of InceptionV3 on this dataset.

**Table 8:** Performance metrics of InceptionV3 model on HFD.

	*P*	*R*	FS
Nonviolence	0.91	0.90	0.91
Violence	0.91	0.92	0.91

### **Performance of the visual geometry group (VGG** )**-19 model**

**Fig. 13:**
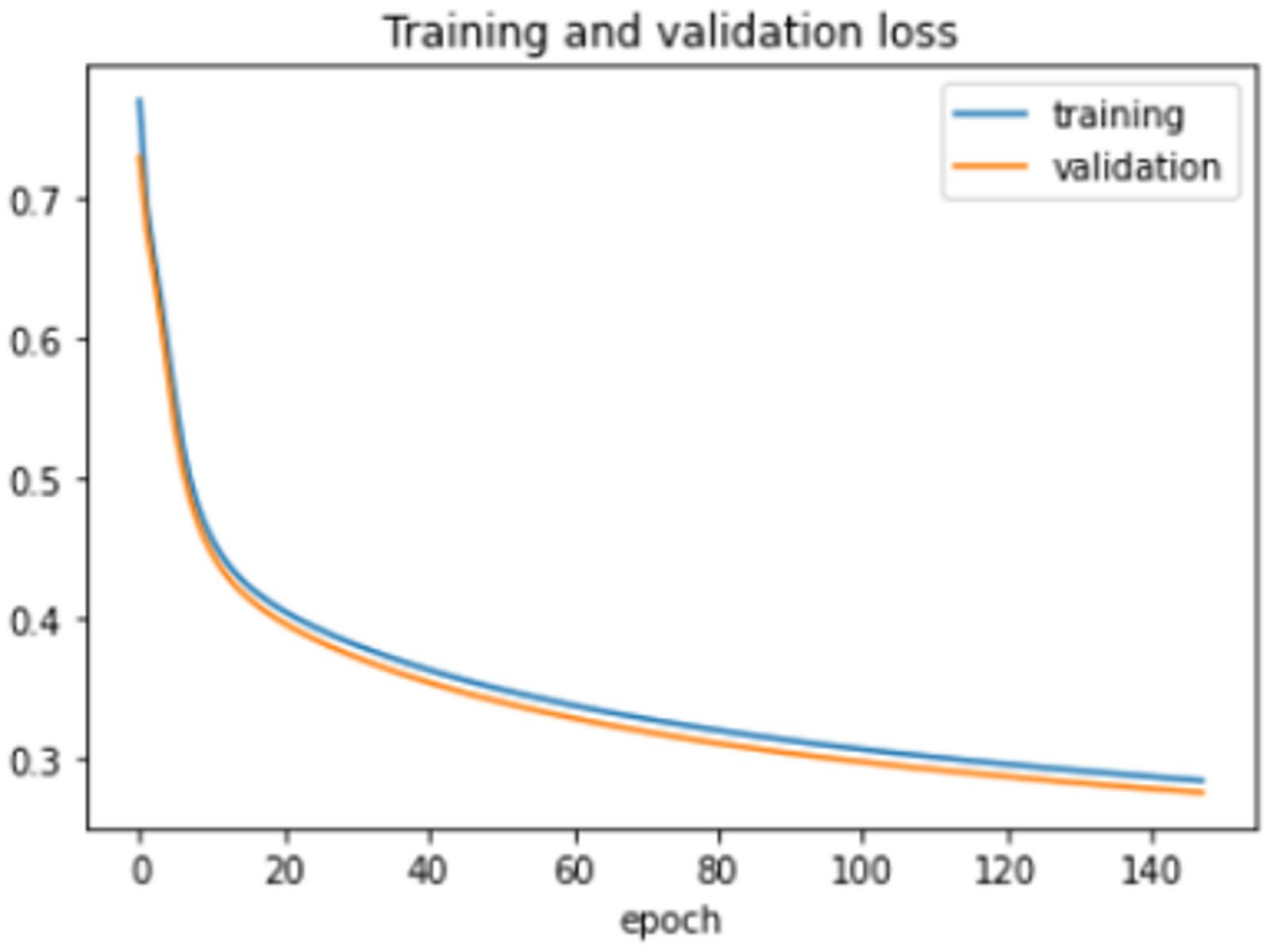
VGG-19’s training loss progress on RLVSD.

**Fig. 14:**
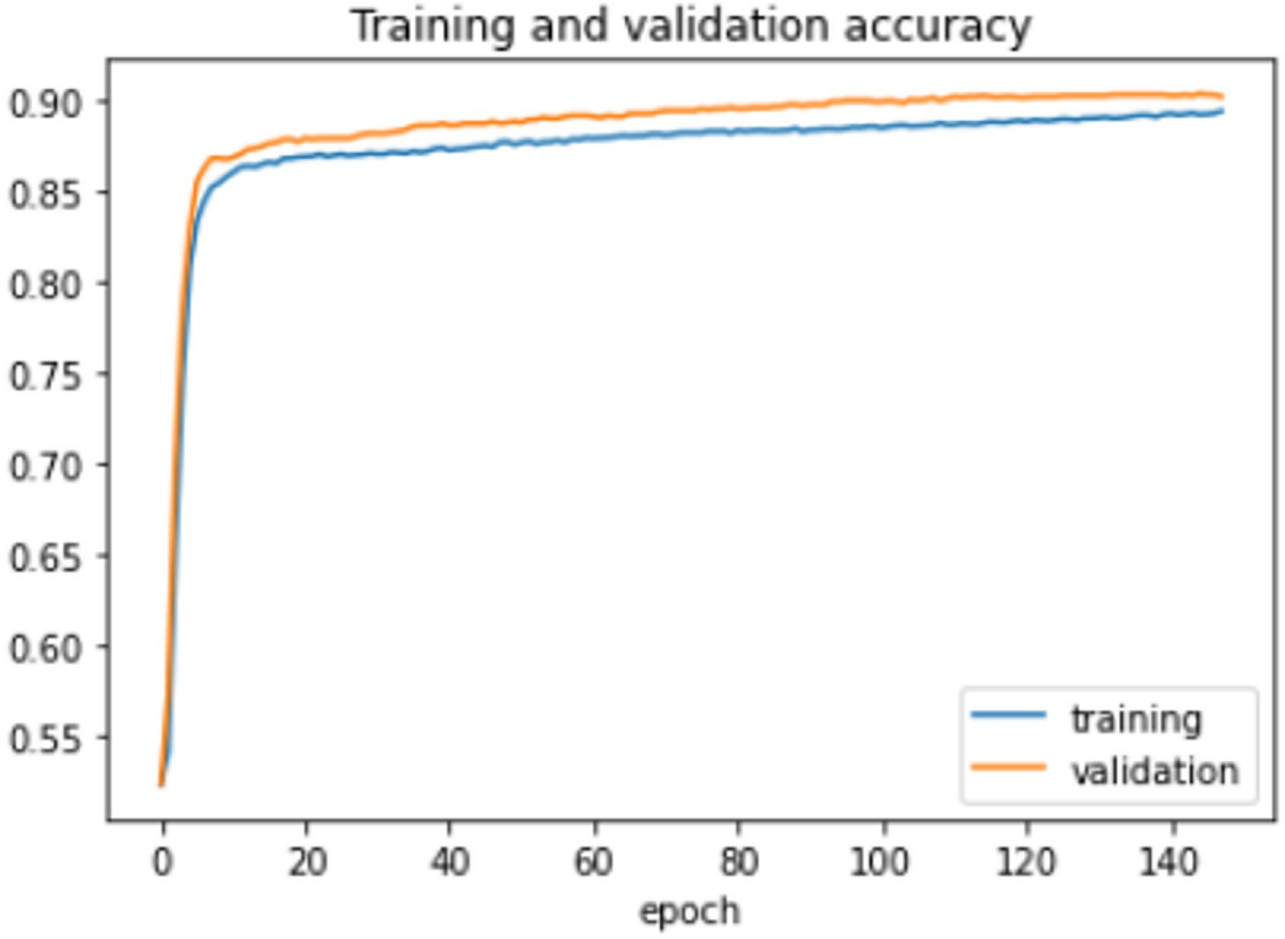
Accuracy progress of the VGG-19 model on RLVSD.

VGG-19 model^43^ consists of 19 convolution layers. It consists of 19 convolution layers, where 16 are convolution layers and 3 are number of fully connected layer. The number of MaxPool layers is 5, and there is one SoftMax Layer. It has 19.6 billion floating-point operations per second. Due to its simplicity, it is very popular and widely used for classification purposes. The VGG-19 model was trained for 150 epochs on the RLVSD with a learning rate of 0.000001. The best epoch was found at 148. The accuracy on the train is 89.43% whereas the accuracy on the test is 90.25%. The loss on the train is 0.2834 and on the test is 0.2745. The other performance metrics of violence and non-violence classes are shown in Table 9. Figure 13 depicts VGG-19’s training loss progress and Fig. 14 depicts the accuracy progress of the VGG-19 model on this dataset.

**Table 9:** Performance metrics of VGG-19 model on RLVSD.

	*P*	*R*	FS
Nonviolence	0.92	0.87	0.90
Violence	0.89	0.93	0.91

**Fig. 15:**
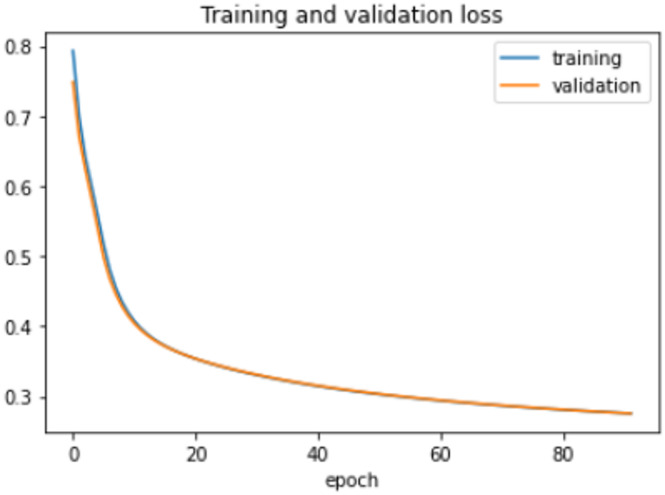
Training loss progress of VGG-19 model on HFD.

**Fig. 16:**
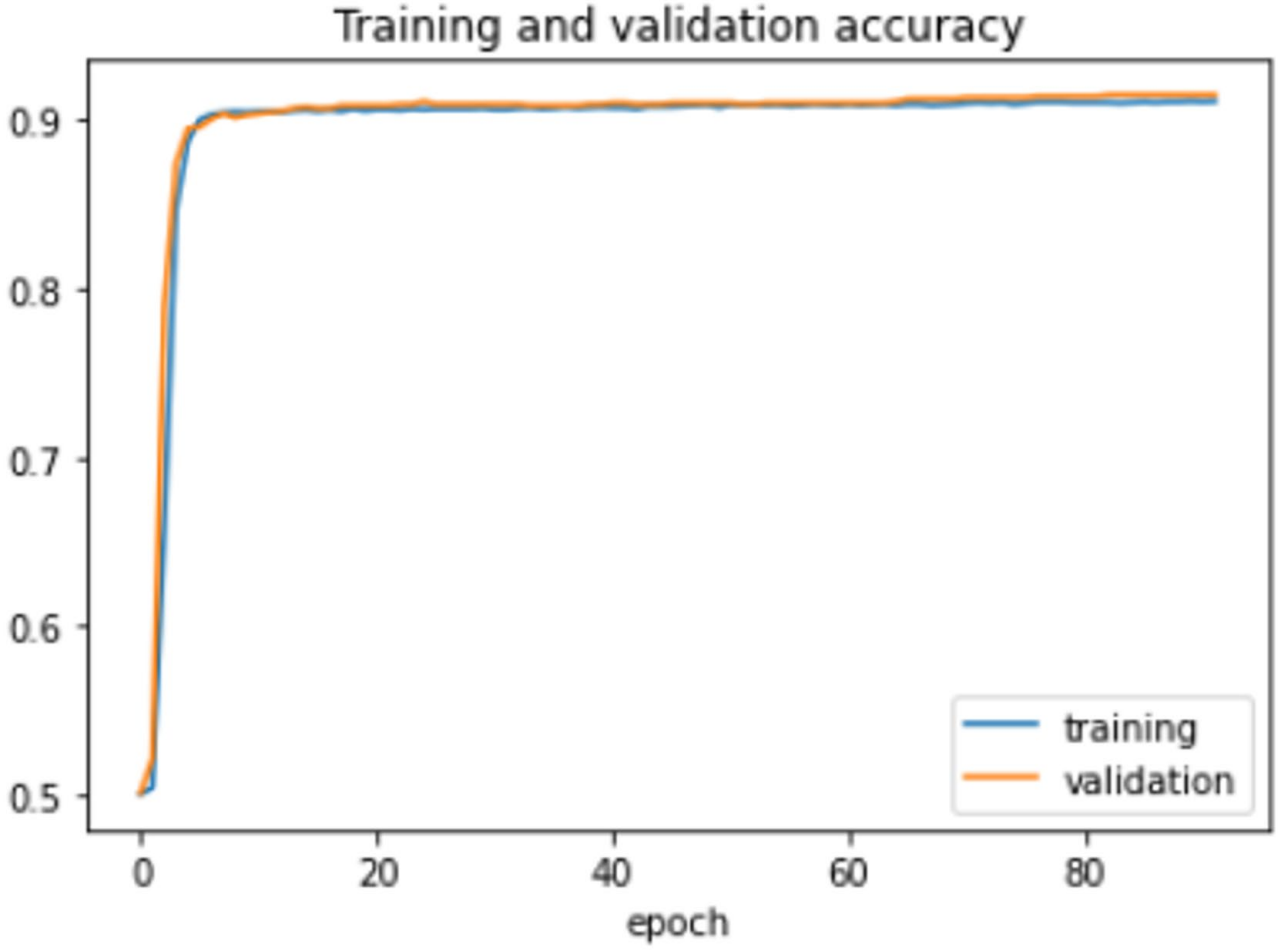
Accuracy progress of theVGG-19 model on HFD.

The VGG-19 model is trained for 110 epochs on the HFD with a learning rate of 0.000001. The best epoch is 92. The accuracy on the train is 91.06% whereas the accuracy on the test is 91.52%. The loss on the train is 0.27504 and on the test is 0.26817. The other performance metrics of violence and non-violence class are shown in Table 10. Figure 15 displays the progress of training loss of VGG-19 on the HFD, while Fig. 16 exhibits the accuracy progression of the same model on the same dataset.

**Table 10:** Performance metrics of VGG-19 model on HFD.

	*P*	*R*	FS
Nonviolence	0.92	0.91	0.91
Violence	0.91	0.92	0.92

### Performance of the MobileNet model

**Fig. 17:**
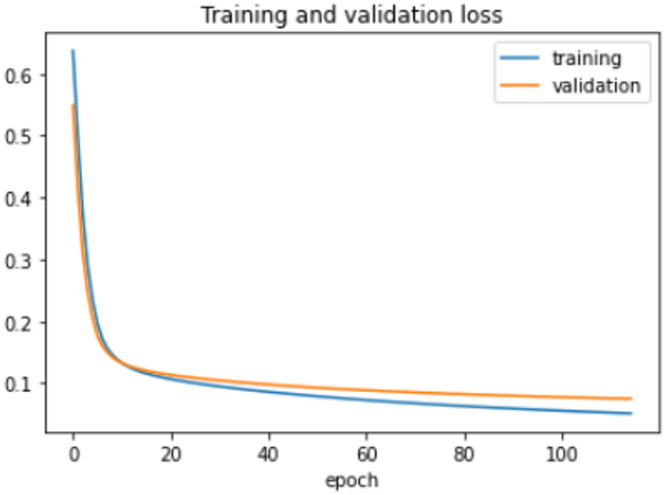
Training loss progress of MobileNet model on RLVSD.

**Fig. 18:**
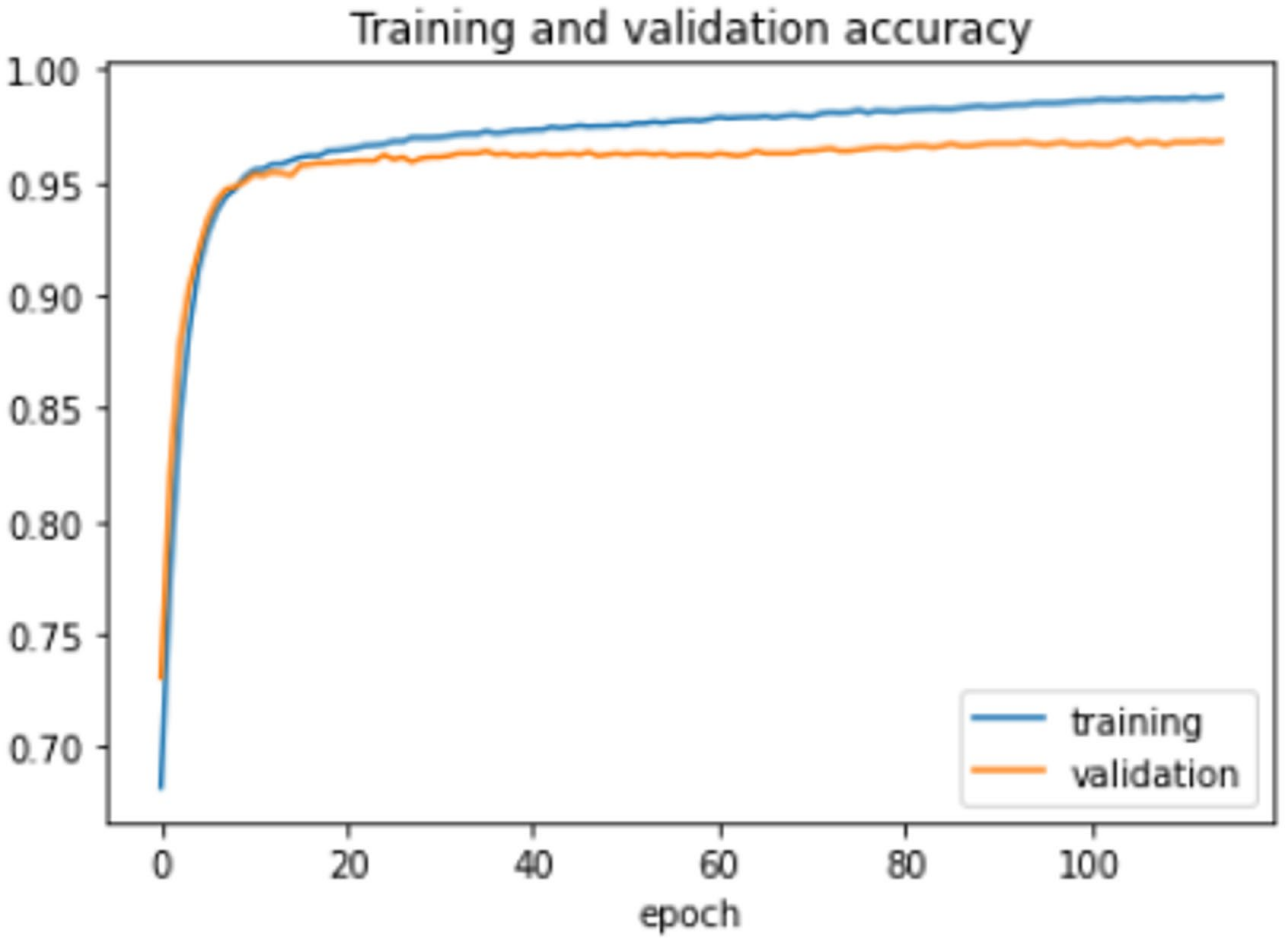
Accuracy progress of the MobileNet model on RLVSD.

MobileNet was proposed by Howard et al.^44^ which can be used in mobile applications. It was a lightweight model with fewer computational parameters. By using depth wise separable convolutions, the parameters of the networks were significantly reduced. It consists of two operations i.e., depth wise convolution and point wise convolution. It has 27 convolution layers of which 13 were depth wise convolution. This model was trained on 150 epochs with a learning rate of 0.000001, using the RLVSD. The accuracy achieved on the non-violent dataset was 97%. Table 11 shows other performance metrics of violence and non-violence class. Figure 17 depicts MobileNet model’s training loss progress and Fig. 18 depicts the accuracy progress of same model on this dataset.

**Table 11:** Performance metrics of MobileNet model on RLVSD.

	*P*	*R*	FS
Nonviolence	0.98	0.96	0.97
Violence	0.96	0.98	0.97

**Fig. 19:**
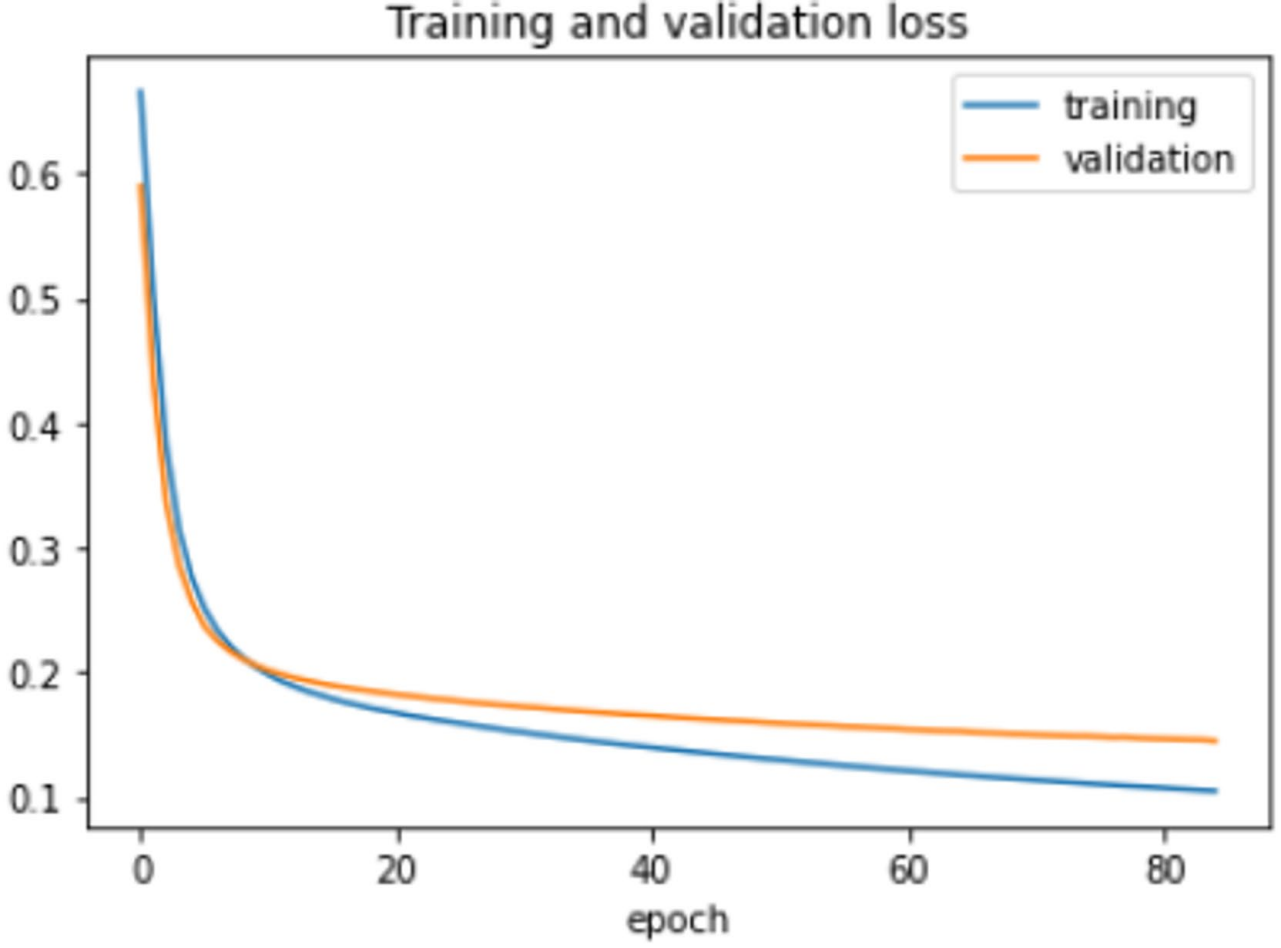
Training loss progress of MobileNet model on HFD.

**Fig. 20:**
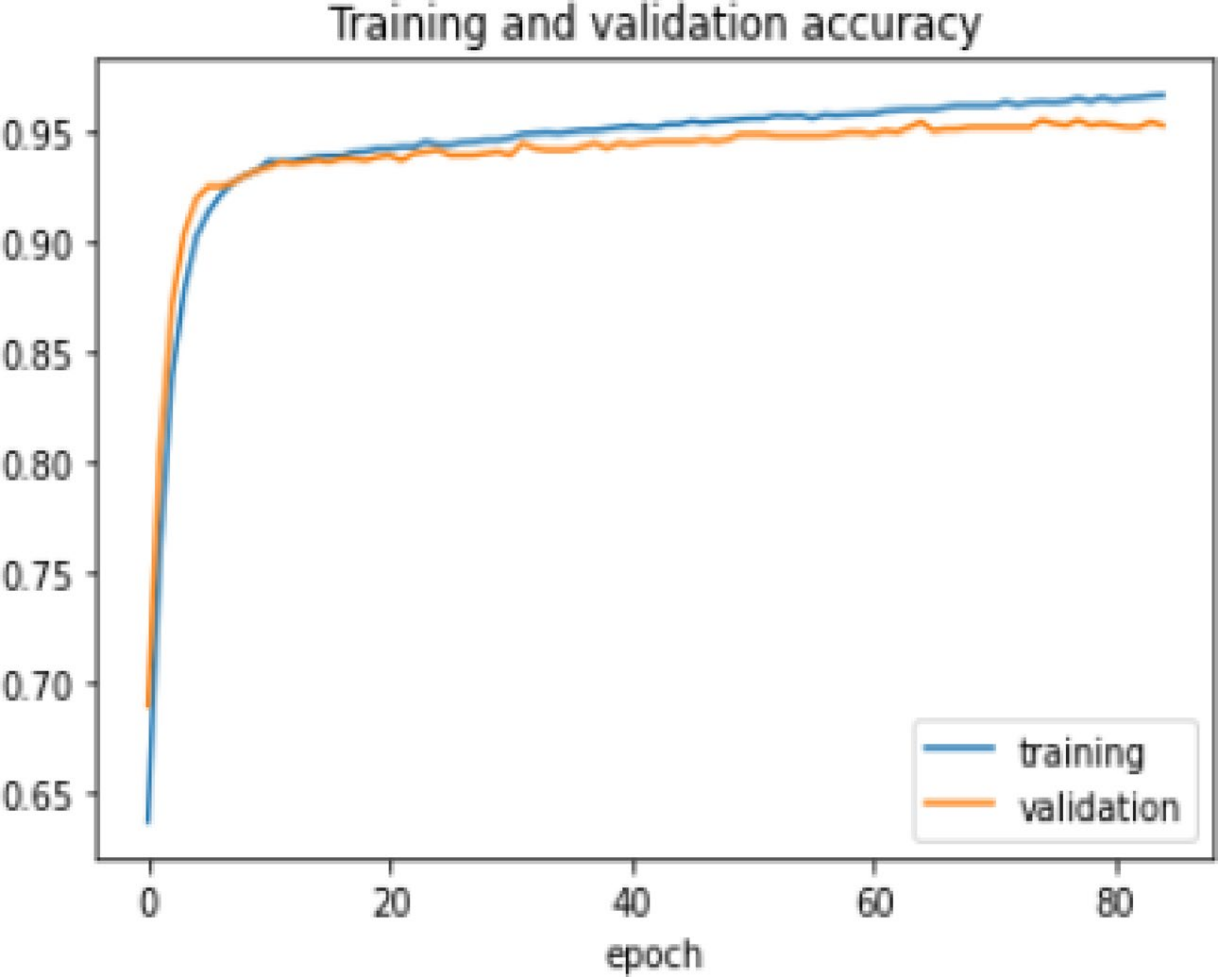
Accuracy progress of the MobileNet model on HFD.

This model is trained for 150 epochs on the HFD with a learning rate of 0.000001. The best epoch is 85. The accuracy on the train is 91.06% whereas the accuracy on the test is 91.52%. The loss on the train is 0.27504 and on the test is 0.26817. The other performance metrics of both the classes are shown in Table 12. A visual illustration of the MobileNet model’s training loss progress can be seen in Fig. 19, while Fig. 20 illustrates the accuracy progress of the same model on HFD.

**Table 12:** Performance metrics of MobileNet model on HFD.

	*P*	*R*	FS
Nonviolence	0.96	0.95	0.95
Violence	0.95	0.96	0.95

### Performance of the Mobilenetv2 model

**Fig. 21:**
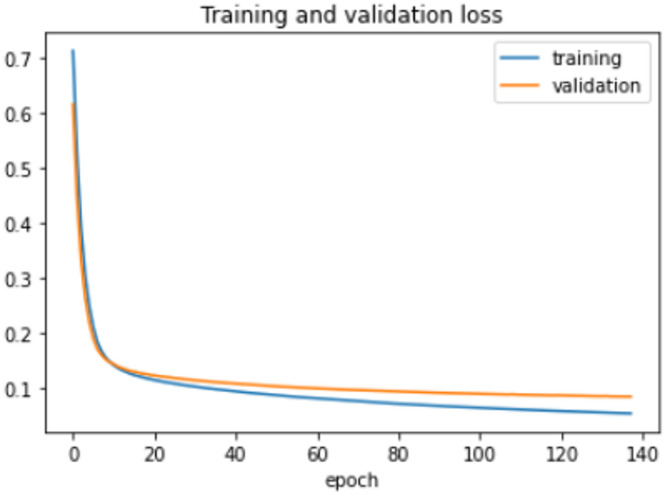
Training loss progress of Mobilenetv2 model on RLVSD.

**Fig. 22:**
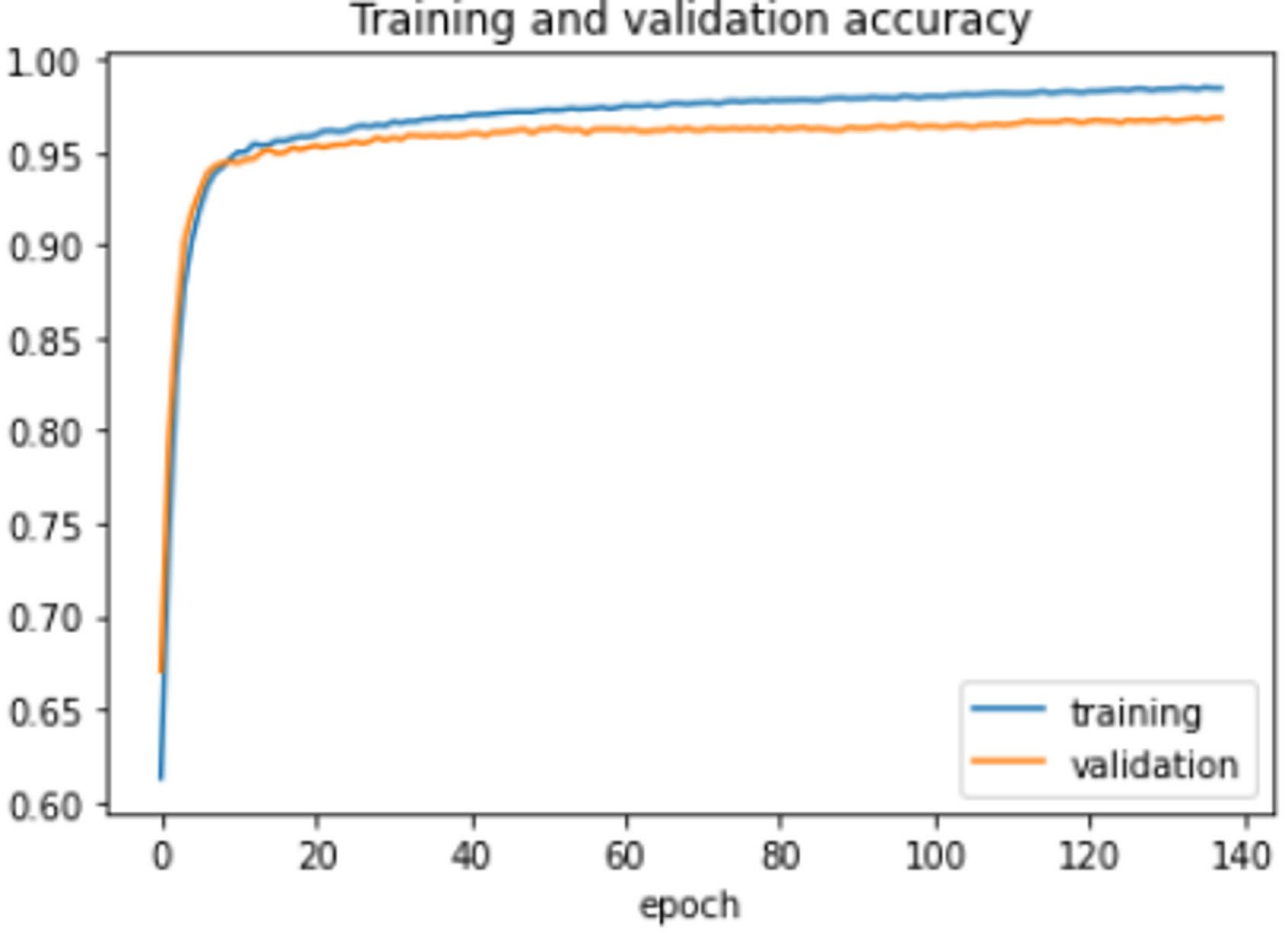
Accuracy progress of the Mobilenetv2 model on RLVSD.

Mobilenetv2 architecture is a lightweight model. It can be used to detect violent activities in videos. This model is lightweight that can be used for embedded and mobile devices where there is a constraint on computational cost and power. It uses pointwise as well as depth wise separable convolution. This model was trained on the RLVSD across 150 epochs with a learning rate of 0.00001. The model’s accuracy for the nonviolence and violence class dataset is 97%. The other performance metrics of both the classes are also recorded in Table 13. Figure 21 illustrates the training loss progress and Fig. 22 displays the accuracy progress of the Mobilenetv2 model on this dataset.

**Table 13:** Performance metrics of Mobilenetv2 model on RLVSD.

	*P*	*R*	FS
Nonviolence	0.97	0.96	0.97
Violence	0.96	0.97	0.97

**Fig. 23:**
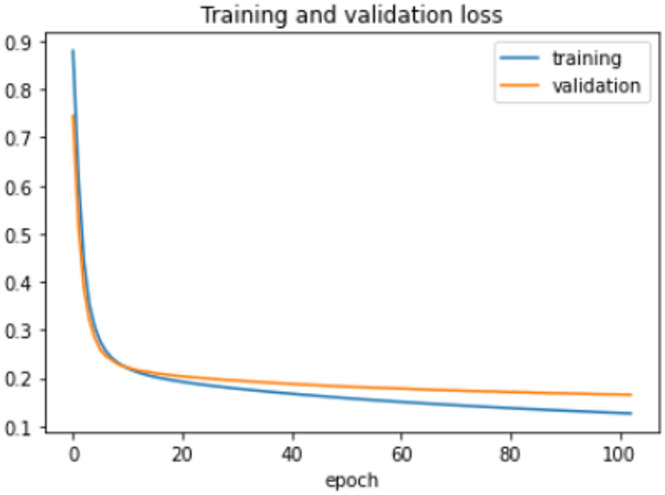
Training loss progress of Mobilenetv2 model on HFD.

**Fig. 24:**
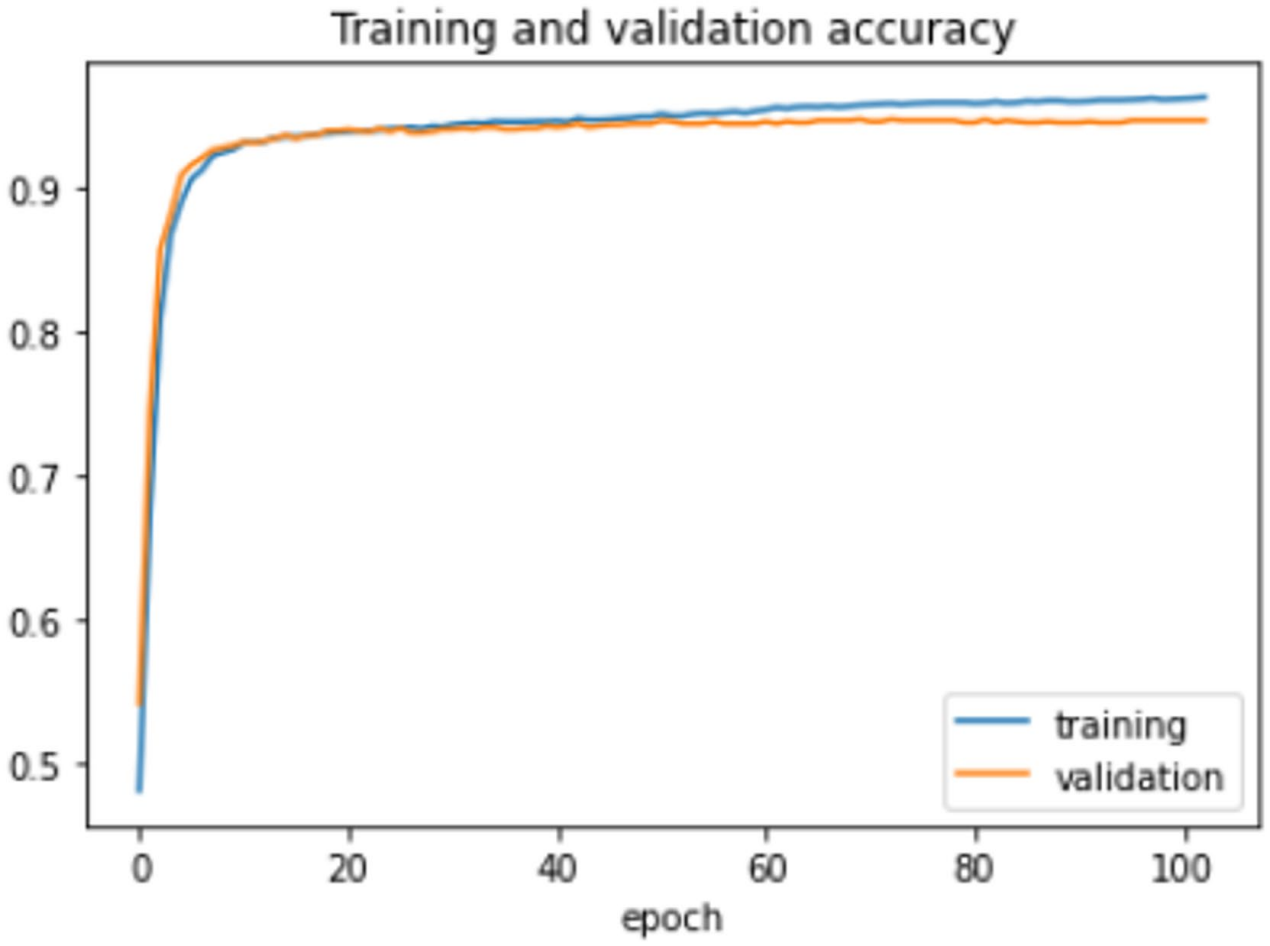
Accuracy progress of the Mobilenetv2 model on HFD.

On the HFD, this model is trained for 150 epochs with the learning rate 0.000001. For non-violence class as well as the violence class, this model attained 95% accuracy. The other performance metrics for the non-violence and violence class are same and recorded in Table 14. Figure 23 illustrates training loss progress and Fig. 24 shows accuracy progress of the model on the Hockey fight dataset.

**Table 14:** Performance metrics of Mobilenetv2 model on HFD.

	*P*	*R*	FS
Nonviolence	0.95	0.95	0.95
Violence	0.95	0.95	0.95

### Performance comparison along with computational parameters

When evaluating the performance of CNN models, the total number of parameters is quite important. Here, we provide a thorough comparative analysis of the proposed model as well as other deep learning models. The Inceptionv3 model has 42 layers. It was proposed by Google in 2015. To decrease the number of computational parameters the factorization of large convolution into smaller is done. It has 22,852,898 computational parameters. It is more efficient in comparison to its previous versions. The VGG-19^43^ model consists of 19 convolution layers. Its computational parameters are 20,024,897. The MobileNet model has 27 convolution layers. Here depth wise separable convolutions were used to reduce the parameters of the networks significantly reduced. The computational parameters of the model are 3,229,889. The Mobilenetv2 model has 53 layers. Its computational parameters are 2,259,265. The proposed approach has a total of 1,937,938 computational parameters.

**Fig. 25:**
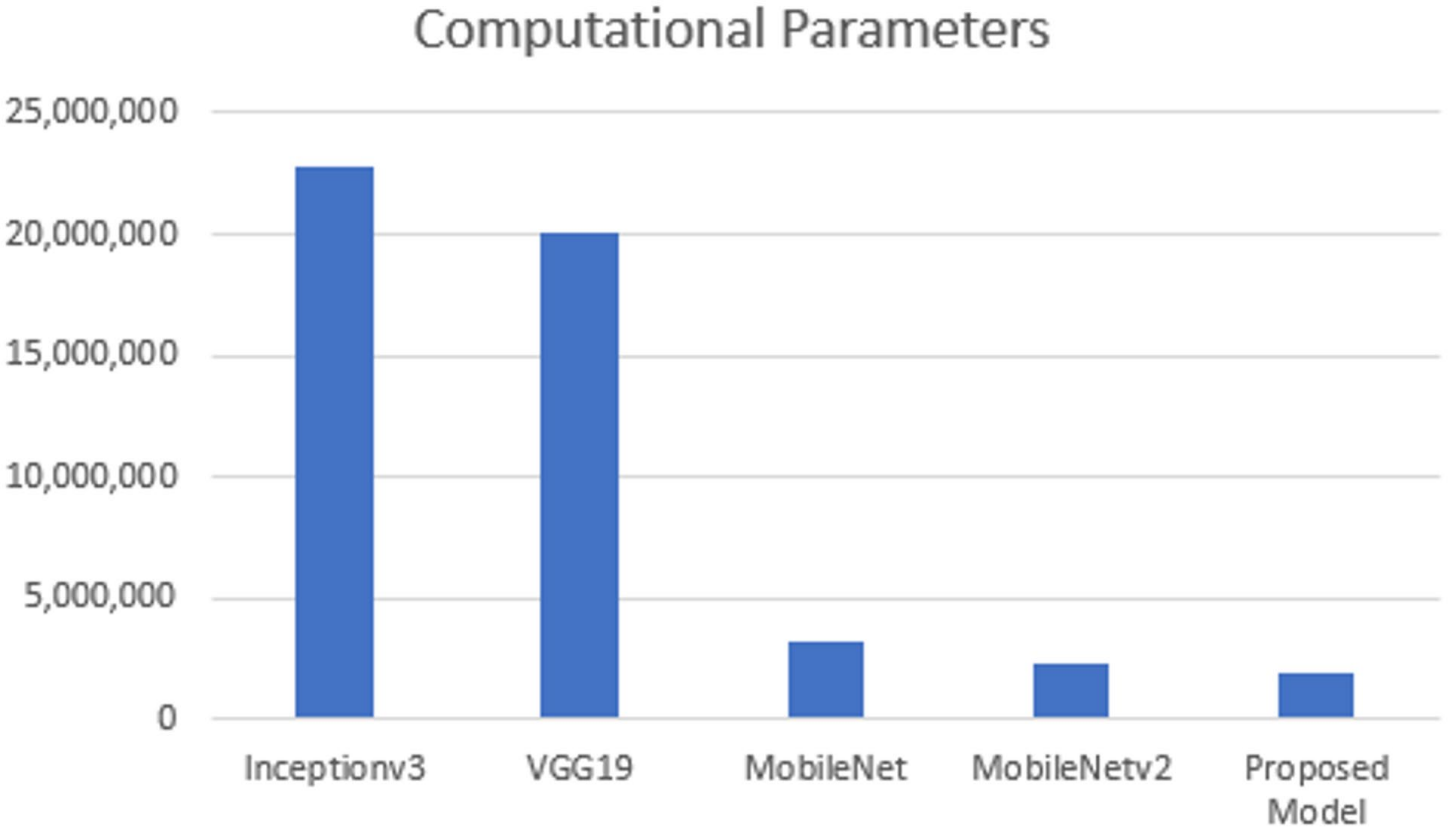
Computational Complexity of InceptionV3, VGG-19, MobileNet, Mobilenetv2 and proposed model.

The visual representation of the comparison of the computational parameters of these models is shown in Fig. 25. It clearly indicates that the proposed model has minimum computational parameters and Inceptionv3 has the highest computational parameters. Table 15 compares the performance of proposed approach to existing state-of-the-art methods on the Real Life Violence Situations dataset. The proposed model reports highest accuracy (97%) which is same as MobileNet and Mobilenetv2. However, the proposed method outperforms with lesser computational parameters. Table 16 shows the performance comparison of proposed method and other state-of-art methods on Hockey fight dataset. In overall, the proposed model reports the second highest accuracy with minimum computational parameters.

**Table 15:** Performance comparison of models on real life violence situations dataset.

Model	Class	*P*	*R*	FS	Accuracy	Parameters
Inceptionv3	Nonviolence	0.95	0.92	0.94	94%	22,852,898
	Violence	0.93	0.96	0.94		
VGG19	Nonviolence	0.92	0.87	0.90	90%	20,024,897
	Violence	0.89	0.93	0.91		
MobileNet	Nonviolence	0.98	0.96	0.97	97%	3,229,889
	Violence	0.96	0.98	0.97		
Mobilenetv2	Nonviolence	0.97	0.96	0.97	97%	2,259,265
	Violence	0.96	0.97	0.97		
Proposed Model	Nonviolence	0.98	0.97	0.97	97%	1,937,938
	Violence	0.97	0.98	0.98		

**Table 16:** Performance comparison of models on hockey fight dataset.

Model	Class	*P*	*R*	FS	Accuracy	Parameters
Inceptionv3	Nonviolence	0.91	0.90	0.91	91%	22,852,898
	Violence	0.91	0.92	0.91		
VGG19	Nonviolence	0.92	0.91	0.91	92%	20,024,897
	Violence	0.91	0.92	0.92		
MobileNet	Nonviolence	0.96	0.95	0.95	95%	3,229,889
	Violence	0.95	0.96	0.95		
Mobilenetv2	Nonviolence	0.95	0.95	0.95	95%	2,259,265
	Violence	0.95	0.95	0.95		
Proposed Model	Nonviolence	0.94	0.94	0.94	94%	1,937,938
	Violence	0.94	0.94	0.94		

In order to comprehensively evaluate the durability of the suggested lightweight architecture, a cross-dataset validation experiment was conducted. As per this evaluation strategy, the model trained on one dataset was put to direct test on the other without going through any additional fine-tuning. This arrangement sets up a situation of a very strong domain-shift since RLVSD and HFD are very different in terms of camera motion, scene structure, illumination, and action dynamics.

When the training was done on RLVSD and testing on HFD, the model showed steady performance indicating its competence in transferring learnt spatiotemporal features to a new place with different action styles and visual characteristics. On the contrary, the training on HFD and testing on RLVSD gave the same consistent results meaning that the model does not rely too much on the specific cues of the dataset. In both cross-dataset testing directions, the architecture has shown a degradation in performance that was predictable and relative to within-dataset performance, this being an expected result under domain-shift conditions, but still maintained the level accuracy that evidences the capability to generalize successfully. The findings suggest that the SE-boosted bottleneck structure and increased factors work together to make the model attentive to the type of motion that is present in different datasets but not to the background textures, lighting, or video source artifacts. Therefore, the cross-dataset results that is given in Table 17 act as supplementary evidence to the fact that the proposed model is able to capture representations that are transferable and ignore the dataset thus being suitable for real-world surveillance deployment.

**Table 17:** Cross-dataset validation results.

Training dataset	Testing dataset	Accuracy (%)	Precision (%)	Recall (%)	F1-Score (%)
RLVSD	HFD	88.6	87.9	86.4	87.1
HFD	RLVSD	90.6	89.8	88.7	89.2

## Conclusion and future scope

In this study, we have developed a framework based on modified Mobilenetv2 for detecting violent behavior in video sequences. The proposed approach demonstrates improved results when measured using quantitative metrics. In addition to this, the proposed model requires minimum computational parameters in comparison to other deep learning models such as Inceptionv3, VGG-19, MobileNet, and MobileNetv2. The proposed method reports 94% accuracy on the Hockey Fight dataset and 97% accuracy on the Real Life Violence Situations dataset. This work is limited to the category of violence detection and focused on fight detection from videos. This model can further be upgraded and tuned to categorize multiple instances of abnormal activities from video sequences for sparse and dense crowd scenes. The use of deep learning-based real-time violence detection systems is a major leap towards securing public safety in different settings like schools, mass transit systems, video surveillance systems, and smart cities. The positive findings of this study demonstrate the capability of such systems to detect violent activity in real-time and with accuracy, enabling instant action by the authorities and hence deterring escalation. A number of areas can be examined for future research. Enhancing the detection accuracy and robustness in occluded or low-light conditions, integration with multi-modal inputs like audio and motion sensors, and minimizing computational expense to deploy on edge devices are areas that require further work. Involving explainable AI methods may also make these systems more transparent and reliable, especially for sensitive deployments. In actual applications, these systems can be wired into CCTV networks for auto-monitoring purposes, applied in transportation centers to trigger security personnel, or installed in schools and hospitals to provide a safer environment. With appropriate ethical considerations and controls in place, real-time violence detection can help bring significant proactive safety efforts and quick emergency response.

## Data Availability

The datasets used and/or analyzed during the current study available from the corresponding author on reasonable request.
